# Re-enforcing hypoxia-induced polyploid cardiomyocytes enter cytokinesis through activation of β-catenin

**DOI:** 10.1038/s41598-019-54334-4

**Published:** 2019-11-28

**Authors:** Yun-Han Jiang, Yu Zhu, Sai Chen, Hai-Long Wang, Yang Zhou, Fu-Qin Tang, Zhao Jian, Ying-Bin Xiao

**Affiliations:** 1 Department of Cardiovascular Surgery, Xinqiao Hospital, Army Medical University, Chongqing, 400037 P. R. China; 2 Department of Cardiothoracic Surgery, The People’s Hospital of Leshan, Leshan, Sichuan Province 614000 P. R. China

**Keywords:** Cardiac hypertrophy, Cardiac regeneration

## Abstract

Cardiomyocyte (CM) loss is a characteristic of various heart diseases, including ischaemic heart disease. Cardiac regeneration has been suggested as a promising strategy to address CM loss. Although many studies of regeneration have focused mainly on mononucleated or diploid CM, the limitations associated with the cytokinesis of polyploid and multinucleated CMs remain less well known. Here, we show that β-catenin, a key regulator in heart development, can increase cytokinesis in polyploid multinucleated CMs. The activation of β-catenin increases the expression of the cytokinesis-related factor epithelial cell transforming 2 (ECT2), which regulates the actomyosin ring and thus leads to the completion of cytokinesis in polyploid CMs. In addition, hypoxia can induce polyploid and multinucleated CMs by increasing factors related to the G1-S-anaphase of the cell cycle, but not those related to cytokinesis. Our study therefore reveals that the β-catenin can promote the cytokinesis of polyploid multinucleated CMs via upregulation of ECT2. These findings suggest a potential field of polyploid CM research that may be exploitable for cardiac regeneration therapy.

## Introduction

Cardiomyocyte (CM) loss is common to several conditions, including ageing and ischaemic heart disease^1^. Although mammalian CMs actively proliferate during embryonic development, this proliferation decreases to a very low level shortly after birth following a shift from a hyperplastic to hypertrophic phenotype^2–4^. Accordingly, the adult mammalian heart has a limited capacity for CM regeneration, and the subsequent replacement of lost tissue with functionally and electrically inert scar tissue leads to cardiac dysfunction and death^5–7^. These characteristics have led to considerable research interest in the potential therapeutic simulation of CM proliferation. Despite decades of cardiac regeneration studies, however, the mechanisms that underlie these regenerative processes remain largely unknown.

CM processes such as hypertrophic growth and DNA synthesis and repair have led many researchers to regard the adult mammalian heart as a pre-mitotic organ in which mitosis remains incomplete despite the presence of some cell cycle proteins and machinery^8^. Within this pre-mitotic state, CMs are forced by multiple pro-hypertrophic factors to re-enter the cell cycle and undergo DNA synthesis in the absence of cytokinesis, leading to polyploidisation and multinucleation^9,10^. Nonetheless, few studies have addressed the limitations of the cytokinesis of polyploid and multinucleated CMs. Most studies have focused on mononucleated CMs, leading to the logical conclusion that new CMs in the adult heart are derived from pre-existing mononucleated CMs. Unfortunately, only a few studies of multinucleated CMs have addressed the relatively reduced proliferative ability of binucleated CMs *in vivo*^11^ or the equivalent ability *in vitro*^12^, compared to their mononucleated counterparts. In contrast to mononucleated CMs, multinucleated polyploid CMs could regenerate substantial amounts of new CMs via karyokinesis and/or cytokinesis, rather than the theoretically more challenging cell cycle re-entry. Hence, an in-depth understanding of the molecular processes that control polyploid and multinucleated CM cytokinesis appears to be of paramount importance.

The Wnt/β-catenin signalling pathway is an essential determinant of cardiac development^13^, including precardiac mesoderm induction and subsequent first heart field formation^14,15^ and second heart field expansion^16^. In this study, we reveal that the activation of β-catenin can force polyploid CM cytokinesis in response to hypoxia. We demonstrate that β-catenin increases the expression of ECT2 to regulate the actomyosin ring, which is essential to the cytokinesis of CM. We also show that hypoxia can regulate cell cycle proteins to induce the formation of polyploid and multinucleated CM. By elucidating the effects of β-catenin on polyploid CM cytokinesis, our study aims to provide information regarding the limitations of polyploid CM cytokinesis and potential therapeutic targets for cardiac regeneration.

## Results

### Increases in ploidy and numbers of nuclei in CMs from patients with cyanotic CHD

**Figure 1:**
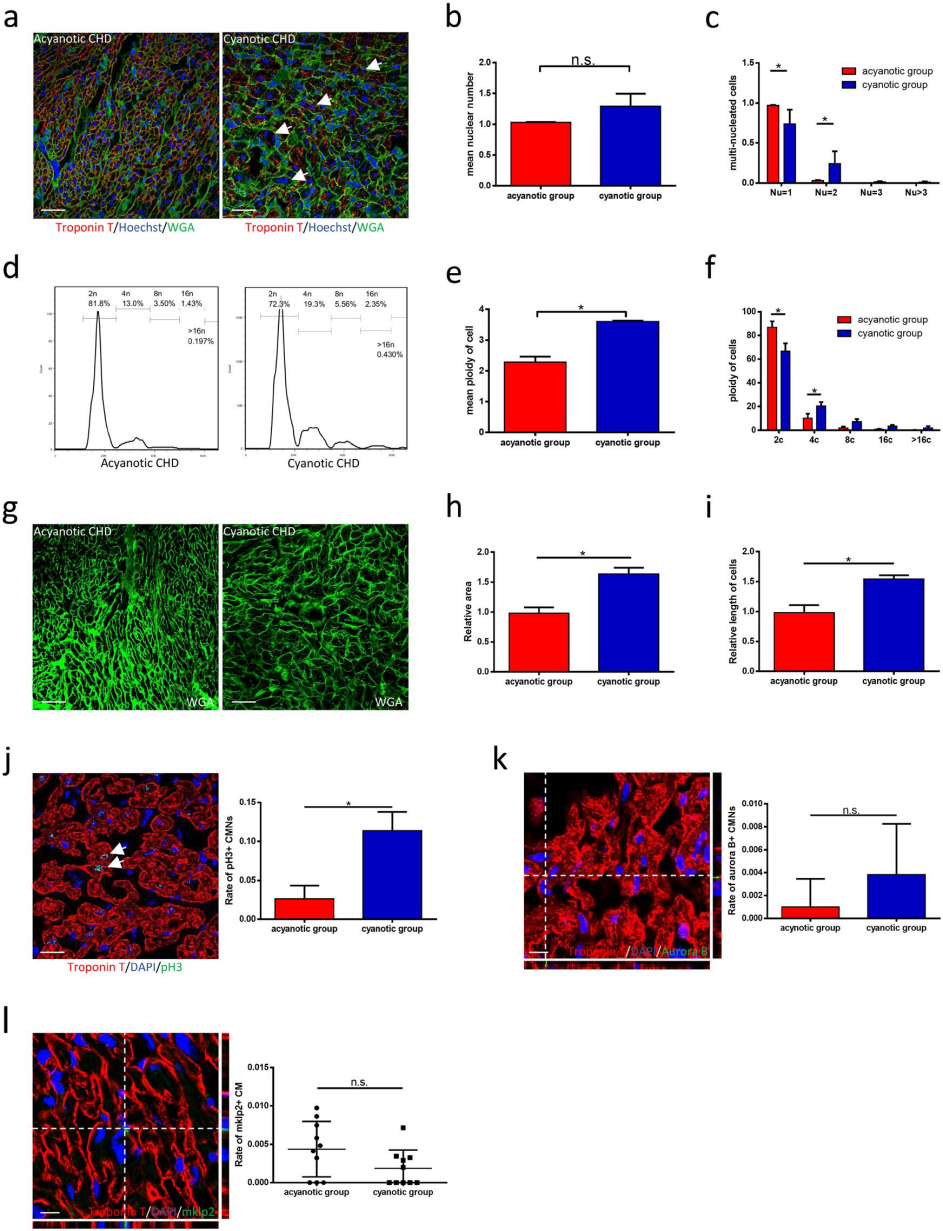
Ploidy and nuclear number of cardiomyocytes is higher in cyanotic congenital heart disease. (**a**) Representative immunofluorescence image of multinucleated cardiomyocytes in acyanotic and cyanotic congenital heart disease. Scale bars, 50 μm. **(b)** The mean nuclear number of cardiomyocytes in both acyanotic and cyanotic group (n = 3 each). **(c)** The percent of multinucleated cardiomyocytes between acyanotic and cyanotic group (n = 3 each). **(d)** Representative distribution of ploidy of cardiomyocytes. **(e)** The mean ploidy of cardiomyocytes (n = 3 each). **(f)** The percent of polyploid cardiomyocytes between acyanotic and cyanotic group (n = 3 each). **(g)** Representative WGA staining in both acyanotic and cyanotic group. Scale bars, 50 μm. **(h)** The result of relative area of cardiomyocytes (n = 5 each). **(i)** The relative length of cardiomyocytes (n = 5 each). **(j)** Co-immunostaining with anti-pH3 and anti-cTnT antibodies. Results are displayed as a presentative image (left, scale bars, 25 μm) and as a bar chart (right, n = 6 each). **(k)** Co-immunostaining with anti-Aurora B and anti-cTnT antibodies. Results are displayed as a representative image (left, scale bars, 10 μm) and as a bar chart (right, n = 6 each). **(l)** Co-immunostaining with anti-mklp2 and anti-cTnT antibodies. Results are displayed as a representative image (left, scale bars, 10 μm) and as a chart (right, n = 10 each). Data is presented as mean ± s.d. *P < 0.05.

To investigate how hypoxia affects ploidy and nuclear numbers in human CMs, we included 10 patients with cyanotic CHD (tetralogy of Fallot) and 10 age-matched patients with acyanotic CHD (ventricular septal defects and right ventricular outflow tract stenosis). The two groups exhibited a large difference in arterial blood oxygen saturation (Supplemental Table S1). The immunofluorescence analysis revealed significantly higher numbers of binucleated CM and a higher mean ploidy in CMs from cyanotic CHD samples relative to acyanotic CHD samples (Fig. 1a–c), as demonstrated by flow cytometry (Fig. 1d–f). Moreover, cyanotic group showed higher mean nuclear ploidy in CM (Supplemental Fig. 3a). An integrated immunofluorescence analysis revealed obvious increases in the numbers of binucleated tetraploid CMs in cyanotic CHD samples (Supplemental Fig. 3b). Correlation analysis revealed arterial oxygen saturation significantly related to mean nuclear ploidy and mean cellular ploidy (Supplemental Fig. 3c,d,e), which implied the possible effect of hypoxia on ploidy. In addition, we observed larger and longer CMs (relative to our standard, Supplemental Fig. 10) in samples from cyanotic patients (Fig. 1g–i). Furthermore, we investigated the CM cell cycle in both groups and observed significant increases in pH3+ CMs (Fig. 1j) in cyanotic tissues, compared to acyanotic tissues, whilst the groups did not exhibit significantly different numbers of aurora B+ CMs (Fig. 1k) and mklp2+ CMs (Fig. 1i). The latter two categories mark the anaphase of mitosis and cytokinesis^17–20^, respectively. Taken together, these results suggest that systematic hypoxia may be responsible for the polyploidisation and multinucleation observed in CMs from cyanotic CHD samples.

### Hypoxia forces CMs to enter an incomplete cell cycle and thus increases ploidy and nuclear numbers both *in vivo* and *in vitro*

**Figure 2:**
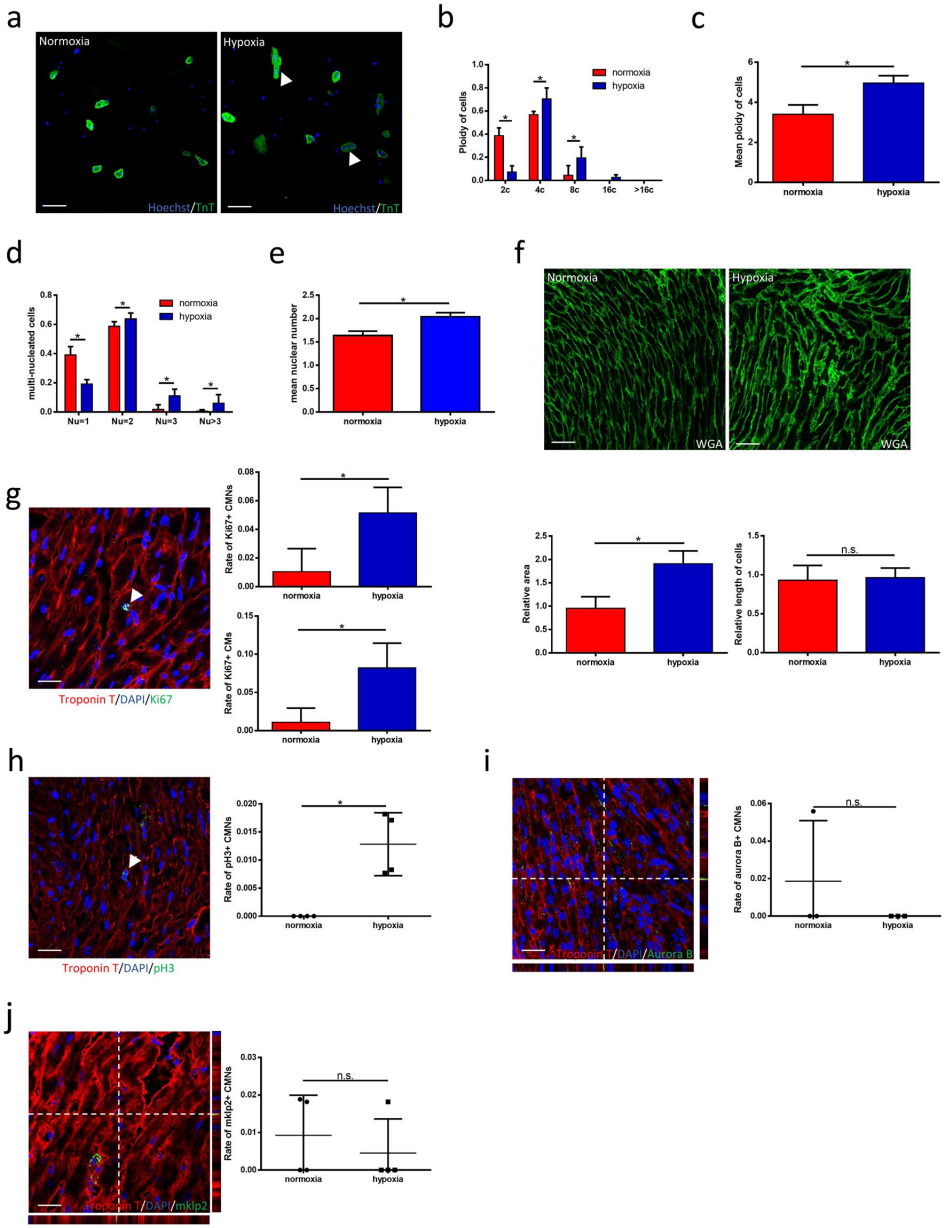
Hypoxia forces CMs to enter an incomplete cell cycle and thus increases ploidy and nuclear numbers *in vivo*. **(a)** Representative distribution of ploidy of cardiomyocytes in both normoxic and hypoxic group (scale bars, 100 μm). The white arrow head indicates trinucleated and tetranucleated cardiomyocytes. **(b)** The percent of polyploid cardiomyocytes between normoxic and hypoxic group (n = 3 each). **(c)** The mean ploidy of cardiomyocytes (n = 3 each). **(d)** The percent of multinucleated cardiomyocytes between acyanotic and cyanotic group (n = 6 each, >600 cardiomyocytes from 10 randomly chosen fields were counted for each individual animal). **(e)** The mean nuclear number of cardiomyocytes in both acyanotic and cyanotic group (n = 6 each, >600 cardiomyocytes from 10 randomly chosen fields were counted for each individual animal). **(f)** WGA staining in both acyanotic and cyanotic group. Results are displayed as two representative images in both group (upper, scale bars, 50 μm), as two bar charts of relative area of cardiomyocytes (lower left, n = 3 each, >600 cardiomyocytes from 10 randomly chosen fields were counted for each individual animal) and relative length of cardiomyocytes (lower right, n = 3 each, >600 cardiomyocytes from 10 randomly chosen fields were counted for each individual animal). **(g)** Co-immunostaining with WGA staining, anti-Ki67 and anti-cTnT antibodies. Results are displayed as a presentative image (left, scale bars, 25 μm), as two bar charts of Ki67-positive cardiomyocyte nuclei (upper right, n = 6 each, >600 cardiomyocytes from 10 randomly chosen fields were counted for each individual animal) and Ki67-positive cardiomyocytes (lower right, n = 3 each, >600 cardiomyocytes from 10 randomly chosen fields were counted for each individual animal). **(h)** Co-immunostaining with anti-pH3 and anti-cTnT antibodies. Results are displayed as a presentative image (left, scale bars, 25 μm) and as a bar chart (right, n = 4 each, >600 cardiomyocytes from 10 randomly chosen fields were counted for each individual animal). **(i)** Co-immunostaining with anti-Aurora B and anti-cTnT antibodies. Results are displayed as a representative image (left, scale bars, 25 μm) and as a bar chart (right, n = 3 each, >600 cardiomyocytes from 10 randomly chosen fields were counted for each individual animal). **(j)** Co-immunostaining with anti-mklp2 and anti-cTnT antibodies. Results are displayed as a representative image (left, scale bars, 25 μm) and as a chart (right, n = 4 each, >600 cardiomyocytes from 10 randomly chosen fields were counted for each individual animal). Data is presented as mean ± s.d. *P < 0.05.

**Figure 3:**
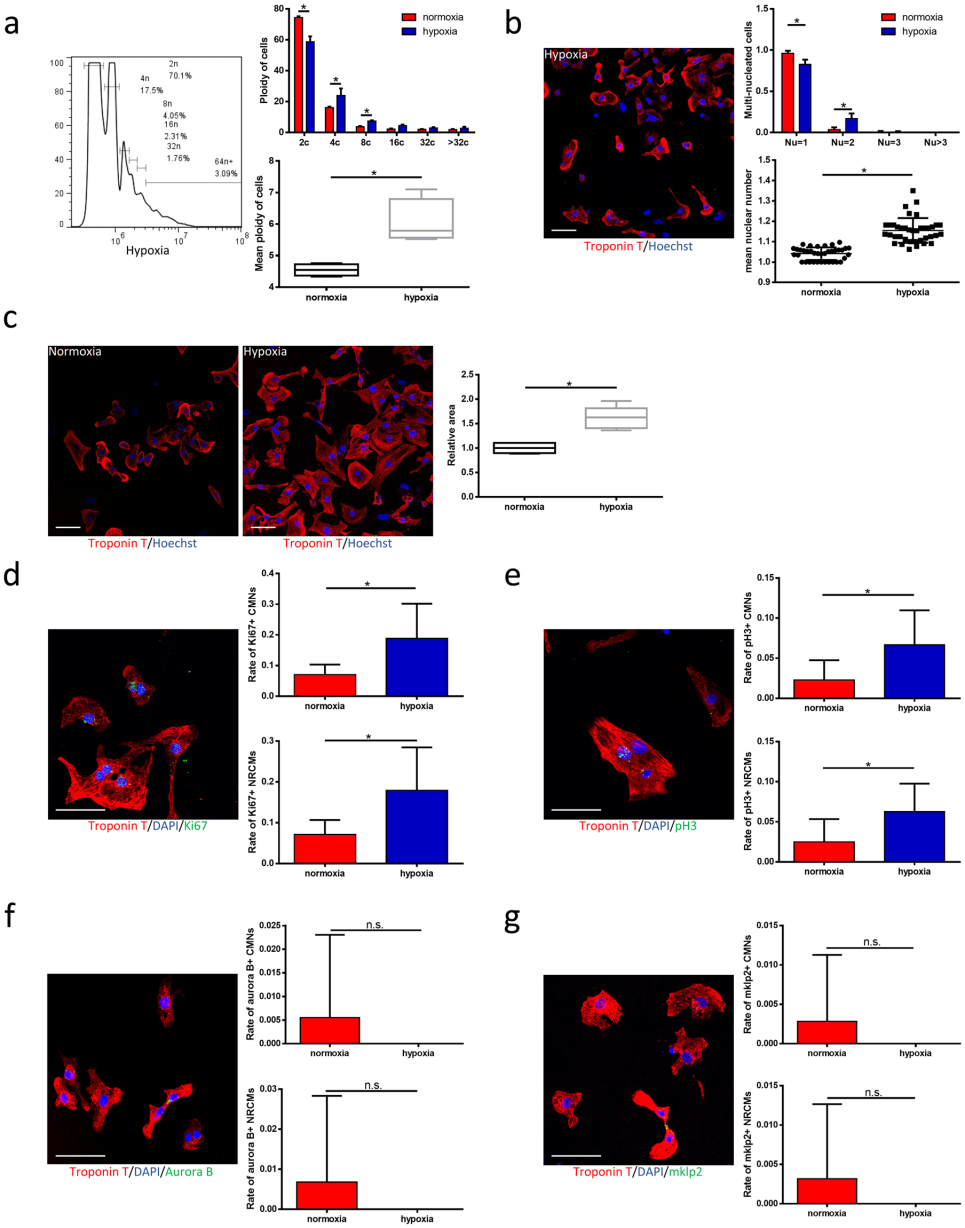
Hypoxia forces CMs to enter an incomplete cell cycle and thus increases ploidy and nuclear numbers *in vitro*. **(a)** Distribution of ploidy of neonatal rat cardiomyocytes (NRCM). Results are displayed as a representative hypoxic cardiomyocytes (left), as a bar chart of ploidy of cardiomyocytes (upper right, n = 4 samples each) and as a box plot of mean ploidy of cardiomyocytes (lower right, n = 4 samples each). **(b)** The nuclear number of NRCMs. Results are displayed as a representative hypoxic image (left, scale bars, 50 μm), as a bar chart of multinucleated cardiomyocytes (upper right, n = 37 samples for normoxia, n = 28 samples for hypoxia, >30 cardiomyocytes from 9 randomly chosen fields were counted for each individual sample) and as a chart for mean nuclear number (lower right). **(c)** Immunofluorescence with anti-cTnT in both normoxic and hypoxic group. Results are displayed as two representative images (left, scale bars, 50 μm) and as a box plot (right, n = 5 samples each, >30 cardiomyocytes from 9 randomly chosen fields were counted for each individual sample). **(d)** Co-immunostaining with anti-Ki67 and anti-cTnT antibodies. Results are displayed as a presentative image (left, scale bars, 100 μm), as two bar charts of Ki67-positive cardiomyocyte nuclei (upper right, n = 6 samples each, >30 cardiomyocytes from 9 randomly chosen fields were counted for each individual sample) and Ki67-positive cardiomyocytes (lower right, n = 6 samples each, >30 cardiomyocytes from 9 randomly chosen fields were counted for each individual sample). **(e)** Co-immunostaining with anti-pH3 and anti-cTnT antibodies. Results are displayed as a presentative image (left, scale bars, 100 μm), as two bar charts of pH3-positive cardiomyocyte nuclei (upper right, n = 9 samples each, >30 cardiomyocytes from 9 randomly chosen fields were counted for each individual sample) and pH3-positive cardiomyocytes (lower right, n = 8 samples each, >30 cardiomyocytes from 9 randomly chosen fields were counted for each individual sample). **(f)** Co-immunostaining with anti-Aurora B and anti-cTnT antibodies. Results are displayed as a representative image (left, scale bars, 100 μm), as two bar charts of Aurora B-positive cardiomyocyte nuclei (upper right, n = 21 samples each, >30 cardiomyocytes from 9 randomly chosen fields were counted for each individual sample) and Aurora B-positive cardiomyocytes (lower right, n = 21 samples each, >30 cardiomyocytes from 9 randomly chosen fields were counted for each individual sample). **(g)** Co-immunostaining with anti-mklp2 and anti-cTnT antibodies. Results are displayed as a representative image (left, scale bars, 100 μm), as two bar charts of mklp2-positive cardiomyocyte nuclei (upper right, n = 19 samples each, >30 cardiomyocytes from 9 randomly chosen fields were counted for each individual sample) and mklp2-positive cardiomyocytes (lower right, n = 19 samples each, >30 cardiomyocytes from 9 randomly chosen fields were counted for each individual sample). Data is presented as mean ± s.d. *P < 0.05.

To demonstrate the effects of hypoxia on CM polyploidisation and multinucleation, mice and isolated CMs were respectively exposed to hypoxia (or normoxia as a control) for 4 weeks and 48 hours. A fluorescence microscope cytometry analysis revealed dramatic increases in the percentages of tetraploid and octoploid CMs and a corresponding decrease in diploid CMs in mice exposed to hypoxia (Fig. 2a–c), consistent with the hypoxic cell model (Fig. 3a). The nuclear numbers in CM were examined via co-immunostaining with anti-TnT, anti-WGA and Hoechst; again, the percentages of binucleated CMs were significantly higher in both hypoxic mice and the cell model, with corresponding decreases in the mononucleated CM fractions (Figs. 2d,e and 3b). The nuclear ploidy of CM is also higher in both hypoxic mice and cell model (Supplemental Figs. 4a–c and 5a,b). Furthermore, there were more mononucleated and bi-nucleated tetraploid CM in hypoxic mice model (Supplemental Fig. 4d) and more bi-nucleated tetraploid NRCM exposed to hypoxia (Supplemental Fig. 5c). In addition, larger CMs were observed in both hypoxic mice and cultures (Figs. 2f and 3c).

As hypoxia induces CM polyploidisation and multinucleation, we further investigated the CM cell cycle during systematic hypoxia. Notably, we observed a significant increase in the percentage of Ki67+ CMs under hypoxia, suggesting an increase in cell cycle activity (Figs. 2g and 3d). Mitosis was measured by immunofluorescence in CMs stained with an anti-pH3 antibody, and the results demonstrated that hypoxia induced more CMs to re-enter the cell cycle (Figs. 2h and 3e). However, hypoxia fail to led to an increased frequency of CMs expressing the anaphase marker Aurora B kinase in both mice and cell cultures (Figs. 2i and 3f). In addition, the hypoxic and normoxic groups did not differ significantly in the expression of the cytokinesis marker mklp2 (Figs. 2j and 3g). Notably, these results were concordant with those obtained from human samples and demonstrate that hypoxia induces CM polyploidisation and multinucleation by forcing the cell to enter mitosis and karyokinesis in anaphase, despite the lack of completed cytokinesis.

### Cell cycle factors account for the major differences in transcription levels between diploid and polyploid CMs

**Figure 4:**
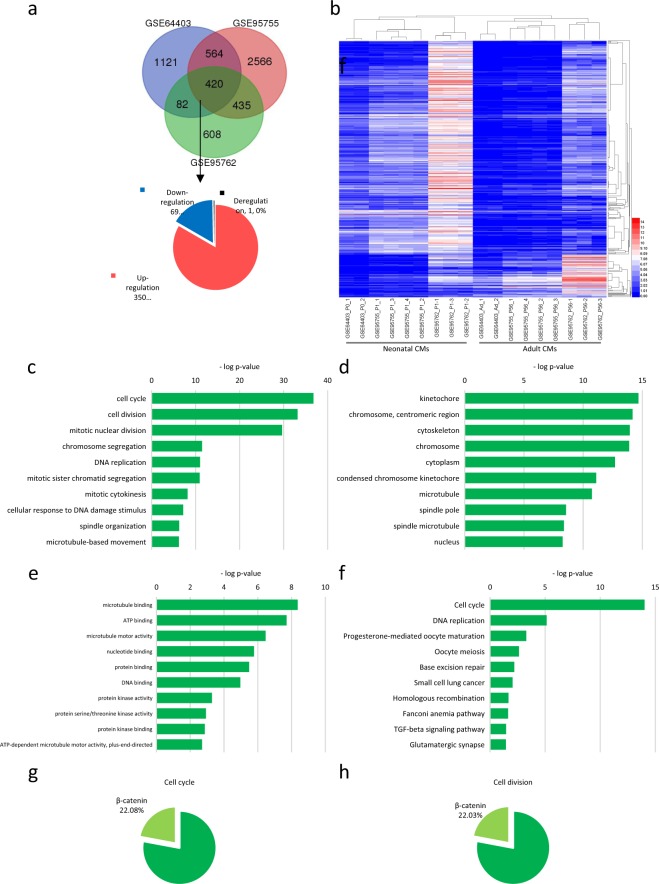
Cell cycle signaling may be the major difference between diploid and polyploid CM. **(a)** Venn plot (upper) and pie chart (lower) of overlapping differentially expressed genes (DEGs) among three datasets. **(b)** Heatmap representation of DEGs across three datasets. **(c)** bar chart of top 10 biological processes of GO terms. **(d)** bar chart of top 10 cellular components of GO terms. **(e)** bar chart of top 10 molecular functions of GO terms. **(f)** bar chart of top 10 KEGG pathways. **(g)** The percentage of β-catenin-regulated genes in cell cycle pathway genes of DEGs. **(h)** The ratio of β-catenin-regulated genes to cell division genes of DEGs.

We next manually searched the GEO and ArrayExpress databases to elucidate the mechanism by which hypoxia induces CM polyploidisation. Although we retrieved 178 datasets, a systematic search identified no directly related microarray data. The indirect data comprised a transgenic mouse and three GEO series (GSE644403, GSE95755, and GSE95762) involving neonatal and adult mice hearts. As transgenes were considered a non-physiological change, data from both neonatal and adult mice were selected for the subsequent microarray analyses. Based on the fact that CM polyploidization during postnatal period, we regarded neonatal mice with hypoploid CM as diploid, while adult mice with hyperploid was defined as polyploid. Comparing the difference between neonatal and adult heart can understand the difference between the diploid and polyploid CM. In a Venn comparison with adult mice, 420 differentially expressed genes (DEGs), including 350 upregulated, 69 downregulated genes, and 1 deregulated gene (upregulated in two datasets and downregulated in the other one) were common in neonates across all three RNA-seq datasets (Fig. 4a). All 420 DEGs in these datasets were visualized using heatmap (Fig. 4b) and classified into the functional categories of biological process, cellular component and molecular function (Fig. 4c–e, respectively) before KEGG pathway analysis (Fig. 4f) with a significance threshold of <0.05. Under biological processes, the most significantly enriched GO terms were observed in the following descending order: ‘cell cycle’ (GO:0007049), ‘cell division’ (GO:0051301) and ‘mitotic nuclear division’ (GO:0007067). Under the cellular components and molecular functions categories, the most enriched GO terms were ‘chromosome’ (GO:0005694) and ‘DNA helicase activity’ (GO:0003678). The most enriched KEGG pathway terms were (in descending order): ‘cell cycle’ (mmu04110), ‘DNA replication’ (mmu03030) and ‘Progesterone-mediated oocyte maturation’ (mmu04914). In addition, a comparison of β-catenin ChIP-seq data revealed that this transcription factor regulated 20.08% of the cell cycle genes (Torre et al, 2011) (Fig. 4g) and 22.03% of cell division genes (Fig. 4h). Overall, these analytical results suggest that diploid and polyploid CMs mainly differ in terms of the regulation of cell cycle and cell division and that β-catenin plays a significant role in this process.

### β-catenin is inactive in cyanotic CHD and hypoxic hearts and NRCMs

**Figure 5:**
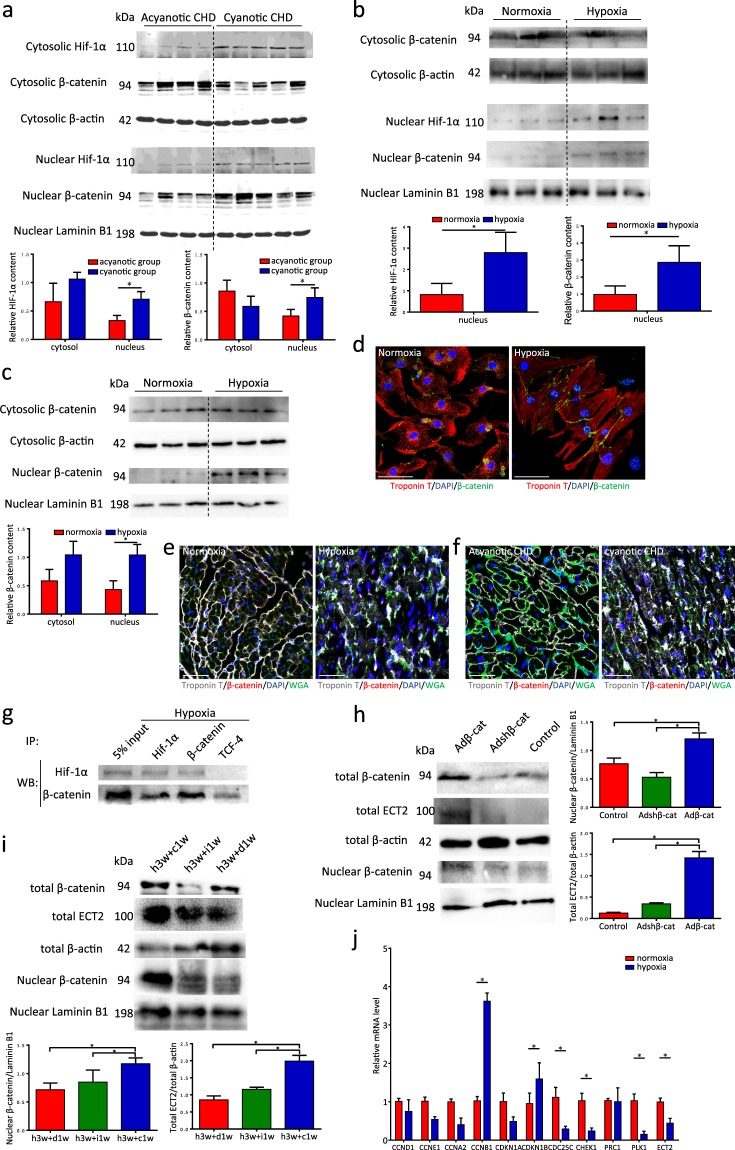
β-catenin is inactive under hypoxia condition. **(a)** Western blot analysis for HIF-1α and β-catenin in nucleus and cytosol in both acyanotic and cyanotic group (n = 4 for acyanotic group, n = 5 for cyanotic group). **(b)** Western blot analysis for HIF-1α and β-catenin in nucleus and cytosol in both mice groups (n = 3 each). **(c)** Western blot analysis for β-catenin in nucleus and cytosol in both NRCM groups (n = 3 sample each). **(d)** Co-immunofluorescence with anti-β-catenin and anti-cTnT antibodies in both NRCM groups. Scale bars, 50 μm. **(e)** Co-immunofluorescence with anti-β-catenin, anti-cTnT antibodies and anti-WGA in both mice groups. Scale bars, 50 μm. **(f)** Co-immunofluorescence with anti-β-catenin, anti-cTnT antibodies and anti-WGA in both acyanotic CHD and cyanotic CHD groups. Scale bars, 50 μm. **(g)** Co-immunoprecipitation for β-catenin, TCF4 and Hif-1. **(h)** Western blot analysis for β-catenin in nucleus and total protein and ECT2 in total protein of NRCMs (n = 3 each). **(i)** Western blot analysis for β-catenin in nucleus and total protein and ECT2 in total protein of mice model (n = 3 each). **(j)** qPCR analysis of cell cycle and cytokinesis genes normalized to actin (n = 3 each). Data is presented as mean ± s.d. *P < 0.05.

Previous studies of mice reported the involvement of β-catenin in embryonic heart development^13^. Accordingly, we investigated β-catenin expression in cyanotic CHD hearts. The nuclear protein content of β-catenin increased in cyanotic CHD samples, although the total protein level did not change significantly (Fig. 5a). A similar phenomenon was observed both *in vivo* and *in vitro* (Fig. 5b–f). To determine whether β-catenin in nuclear have the transcription activity, the downstream gene was detected by qPCR. The downstream gene CCND1 had non-significant change between hypoxic and normoxic mice model (Fig. 5g). This result suggested the inactive state of β-catenin, although β-catenin relocated from cytoplasm into nucleus.

To identify the reason of the contradictory results of β-catenin, we performed co-immunoprecipitation to examined interaction among β-catenin, TCF4 and Hif-1. There was more β-catenin/Hif-1α binding than β-catenin/TCF4 under hypoxia (Fig. 5g), and reverse co-IPs support these data (Fig. 5g). These results suggested that the nuclear location of β-catenin mainly bound to Hif-1 not the transcription factors TCF4 and hif-1 hampers the binding of β-catenin and TCF4 via competitive inhibition under hypoxia.

### Active β-catenin decreases the ploidy and nuclear number of CMs *in vitro* and *in vivo*

**Figure 6:**
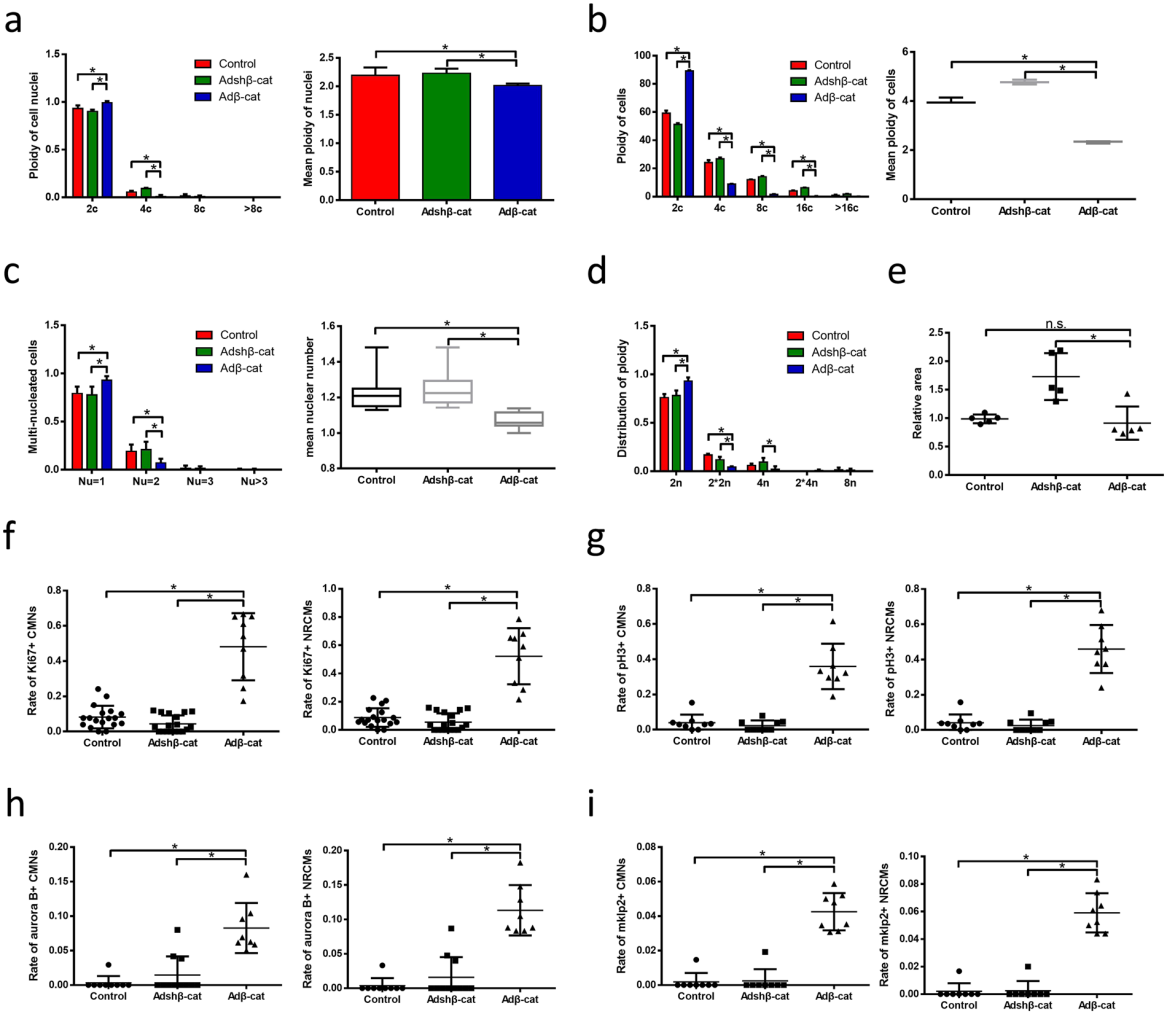
Active β-catenin reduce ploidy and nuclear number of neonatal rat cardiomyocytes. **(a)** Distribution of ploidy (left, n = 5 samples each) and mean ploidy (right, n = 5 samples each) of neonatal rat cardiomyocyte nuclei via flow cytometry. **(b)** Distribution of ploidy (left, n = 3 samples each) and mean ploidy (right, n = 3 samples each) of NRCMs. **(c)** Distribution of ploidy (left, n = 12 samples each) and mean ploidy (right, n = 12 samples each) of NRCMs. **(d)** Distribution of NRCM ploidy (n = 3 samples each). **(e)** Relative area of NRCMs (n = 5 samples each). **(f)** The percent of Ki67-positive cardiomyocyte nuclei (left, n = 9 samples each) and Ki67-positive cardiomyocytes (right, n = 9 samples each). **(g)** The percent of pH3-positive cardiomyocyte nuclei (left, n = 8 samples each) and pH3-positive cardiomyocytes (right, n = 8 samples each). **(h)** The percent of Aurora B-positive cardiomyocyte nuclei (left, n = 8 samples each) and Aurora B-positive cardiomyocytes (lower right, n = 8 samples each). **(i)** The percent of mklp2-positive cardiomyocyte nuclei (upper right, n = 8 samples each) and mklp2-positive cardiomyocytes (lower right, n = 8 samples each). Data is presented as mean ± s.d. *P < 0.05.

**Figure 7:**
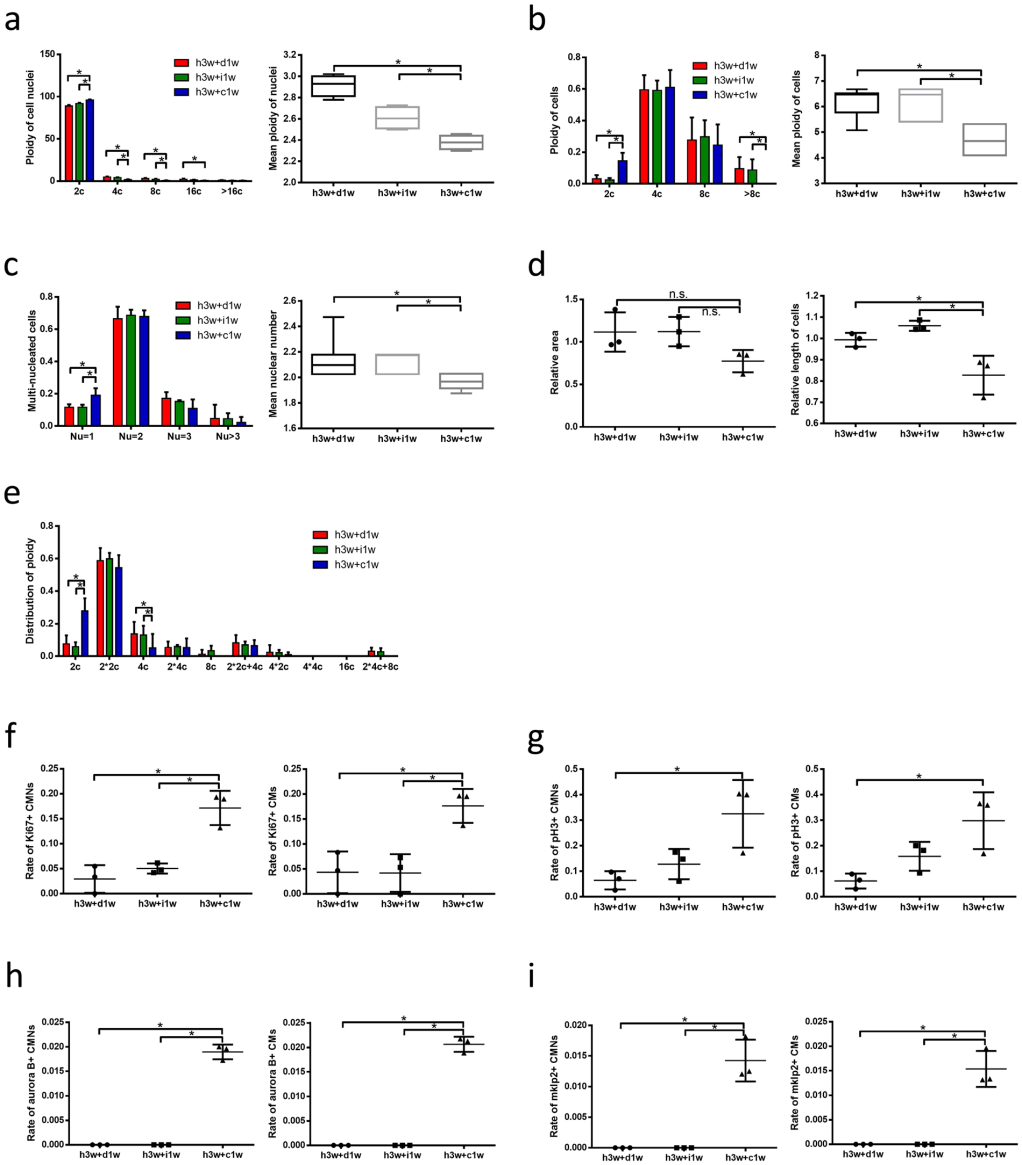
Active β-catenin reduce ploidy and nuclear number of cardiomyocytes *in vivo*. **(a)** Distribution of ploidy (left, n = 4 each) and mean ploidy (right, n = 4 each) of cardiomyocyte nuclei via flow cytometry. **(b)** Distribution of ploidy (left, n = 4 each) and mean ploidy (right, n = 4 each) of cardiomyocytes. **(c)** Distribution of ploidy (left, n = 15 each) and mean ploidy (right, n = 15 each) of cardiomyocytes. **(d)** Relative area (left, n = 3 each) and relative length (right, n = 3 each) of cardiomyocytes. **(e)** Distribution of cardiomyocyte ploidy (n = 3 each). **(f)** The percent of Ki67-positive cardiomyocyte nuclei (left, n = 3 each) and Ki67-positive cardiomyocytes (right, n = 3 each). **(g)** The percent of pH3-positive cardiomyocyte nuclei (left, n = 3 each) and pH3-positive cardiomyocytes (right, n = 3 each). **(h)** The percent of Aurora B-positive cardiomyocyte nuclei (left, n = 3 each) and Aurora B-positive cardiomyocytes (lower right, n = 3 each). **(i)** The percent of mklp2-positive cardiomyocyte nuclei (upper right, n = 3 each) and mklp2-positive cardiomyocytes (lower right, n = 3 each). Data is presented as mean ± s.d. *P < 0.05.

To determine the potential role of β-catenin in polyploid CM cytokinesis, we evaluated the direct effects of β-catenin overexpression or silencing on ploidy and the nuclear number of NRCMs *in vitro*. β-catenin overexpression enhanced the transcription activity of this protein, as confirmed by a western blotting analyse (Fig. 5e) and qPCR analysis of downstream genes (Supplemental Fig. 1g). Notably, both ploidy and the nuclear number were very clearly decreased in NRCMs subjected to overexpression, compared to those subjected to silencing or treated with vehicle alone (Fig. 6a–d). *In vivo*, the ploidy distribution and nuclear number were consistent with those observed in NRCMs (Fig. 7a–c,e). These data suggest that β-catenin may promote the completion of polyploid multinucleated CM cytokinesis.

Regarding cell cycle activity, we observed a significant increase in Ki67+ CMs in the β-catenin overexpression group (Fig. 6f), as well as higher percentages of pH3+ (Fig. 6g), aurora B+ (Fig. 6h) and mklp2+ CMs (Fig. 6i), suggesting that β-catenin induces CMs to enter the cell cycle and undergo karyokinesis and cytokinesis. Similarly, *in vivo* experiments demonstrated obvious increases in Ki67, pH3, aurora B and mklp2 expression in the CHIR99021 treatment group (Fig. 7f–i). These findings indicate that β-catenin reduces the ploidy and nuclear number of CMs exposed to hypoxia and may induce the cytokinesis of hypoxia-induced polyploid multinucleated CMs.

### β-catenin promotes tetraploid CM cytokinesis by promoting the expression of the cytokinesis factor ECT2

To confirm the effect of β-catenin on polyploid CM cytokinesis, we sorted hypoxia-induced tetraploid CMs via flow cytometry and treated the cells with CHIR99021. A flow cytometric analysis revealed a reduction in CM ploidy during hypoxia in the presence of CHIR99021 (Supplemental Fig. 1k,l). Moreover, the number of CMs increased significantly in active β-catenin of both cell and mice model (Supplemental Fig. 6a–d), which also confirmed the effect of β-catenin on cytokinesis.

To elucidate the mechanism by which β-catenin promotes polyploid CM cytokinesis, we attempted to clarify the role of β-catenin in cytokinesis regulation. Because β-catenin is mainly localised at the centrosome during mitosis^21,22^, β-catenin–mediated regulation may primarily involve the transcription of factors related to cytokinesis. We performed qPCR to detected the mRNA level of cytokinesis-related genes (*PRC1*, *PLK1*, *ECT2*) selected from literature research (Fig. 5j, Supplemental Fig. 1j). These data reveals PLK1 and ECT2 may play a vital role in CM cytokinesis. Analysing the promoter of these genes, we identified two potential β-catenin-regulated site located at the promoter and intron of *ect2* gene of mice and rat (Supplemental Fig. 1a), which is required for both cytokinesis and multinucleation during embryonic development^23^. We then applied chromatin immunoprecipitation (ChIP)-qPCR assay to confirm our hypothesis. The results showed β-catenin occupancy of the motif 1 in CHIR99021 group of NRCM and mice, but the occupancy was greatly reduced in another motif (Supplemental Fig. 1b,c). These findings supported that β-catenin directly regulates *ect2* and induces its transcription in CMs.

Based on this evidence, we further evaluated the effect of ECT2 on CM cytokinesis using immunofluorescence assays. ECT2 is a scaffold protein that interacts with multiple cytoskeletal proteins and regulators to promote constriction of the actomyosin ring and phosphorylation of the small G protein RhoA-GDP and thus the regulation of anillin localisation^24^ (Supplemental Fig. 9). Anillin is a cytoskeletal factor involved in actomyosin ring constriction, and defects in the localisation of this factor cause cytokinesis failure and CM binucleation^25^. In this study, β-catenin activation improved the localisation of anillin and ECT2 (Supplemental Fig. 1d–f). Knocking down of ECT2 significantly increases the ploid and nuclear number of cardiomyocytes (Supplemental Fig. 13a–c), and reduces the percentage of mklp2+ CM only in cell cycle activity experiments (Supplemental Fig. 13d–g). Furthermore, RhoA-GTP is dephosphorylated to RhoA-GDP by MgcRacGAP, another regulator of cytokinesis^24^. Also, the dislocation of MgcRacGAP was obvious in the bi-nucleated CMs (Supplemental Fig. 1g). Our Immunofluorescence analysis revealed a significantly higher frequency of MgcRacGAP localisation in the mid-body (Supplemental Fig. 1h,i). Therefore, β-catenin promotes hypoxia-induced tetraploid CM cytokinesis by upregulating ECT2, which in turn promotes anillin and MgcRacGAP localisation in the actomyosin ring.

### β-catenin improves cardiac function without increasing hypoxia-induced angiogenesis and inhibiting CM apoptosis

Tissue regeneration is usually accompanied by angiogenesis^26^. Therefore, we evaluated the effects of active β-catenin on the size and density of capillaries in hypoxic hearts. Notably, CHIR99021-treated hearts failed to exhibited increases in both capillary size and density, compared to hearts from the IWR-1 and DMSO groups (Supplemental Fig. 2a). We also used TUNEL staining to evaluate the apoptosis of CMs, particularly polyploid cells. We fail to observed any significant decrease of TUNEL-positive CMs in hearts and NRCMs exhibiting β-catenin activity (Supplemental Fig. 2b,c). In addition, a haemodynamics analysis revealed a significantly higher left ventricle ejection fraction amongst mice in the CHIR99021 group, compared to those in the IWR-1 and DMSO groups (Supplemental Fig. 2d,e). Other detail cardiac function index had been showed in Supplemental Table S4 and S5. Together, these results indicate that active β-catenin improves cardiac function without promoting angiogenesis and inhibiting CM apoptosis.

## Discussion

In this study, we have demonstrated that β-catenin activation directs hypoxia-induced polyploid CMs to undergo cytokinesis and have identified the following major novel findings: (1) hypoxia significantly induces CM polyploidisation and multinucleation; (2) major differences in transcription levels between diploid and polyploid CMs are mainly attributable to cell cycle-related factors and (3) active β-catenin directs polyploid CMs to undergo cytokinesis and divide into new daughter CMs. Collectively, these findings suggest that β-catenin plays a pivotal role in the cytokinesis of polyploid CMs. To the best of our knowledge, this study is the first to address different polyploid CM subtypes (rather than binucleated CMs alone) and to demonstrate that hypoxia-induced polyploid CMs also retain the potential for cytokinesis, which can be induced by β-catenin activation. Furthermore, this study demonstrates the potential of polyploid CMs as a new research and therapeutic target for cardiac regeneration.

In vertebrate species, hypoxia is an intriguing pathophysiological condition that shares a complex link with the proliferative capacities of CMs throughout the lifespan. Fish and amphibians, in which arterial and venous blood mixing occurs^27^, exhibit a remarkable capacity for cardiac regeneration throughout their lifespans^28–31^. Similarly, in various mammalian embryonic models, arteriovenous mixing leads to an arterial partial oxygen pressure of 25 to 35 mm Hg^32–37^ and the consequent ability to regenerate injured cardiac tissue^38,39^. However, mammals quickly lose this regenerative capacity after birth, partially because of a rapid rise in the partial oxygen pressure to approximately 100 mm Hg^40^. These earlier findings confirmed the role of hypoxia as a necessary and sufficient factor for cardiac regeneration in several species. However, the incomplete regeneration capacity of neonatal mammals with normoxic blood remains somewhat unclear and suggests that the effect of hypoxia on this capacity may be limited in adult mammals. In addition, multiple studies have reported various pathological and physiological factors in the regulation of CM polyploidisation and multinucleation in adult mammals^41–45^, suggesting that CMs could re-enter the cell cycle and undergo DNA synthesis despite an inability or limited ability to achieve cytokinesis. Similarly, our work has demonstrated the powerful ability of hypoxia to stimulate DNA synthesis and, partly, karyokinesis in CMs, and we have confirmed this ability in human samples, mouse models and NRCMs. Our work has further confirmed that hypoxia is not sufficient to induce cardiac regeneration in adults. Other group found reduced nuclear number of cardiomyocytes under an extreme hypoxic environment (7% O2)^46^. In their study, adult mice were exposed to a gradual (1% per day) decrease and were kept at 7% oxygen for 2 weeks. Our previous study showed different oxygen concentration affect cardiac function^47^, suggesting that distinct hypoxic environment may activate the G1-S phase or ana-telophase of M phase of CMs, leading to polyploidization or regeneration. Further research needs to find the molecular mechanism in different effect of hypoxic condition on the cycle activity of CMs.

Polyploid and multinucleated CMs are an extremely common factor in various heart diseases, including CHD and hypertensive heart disease^48^. Generally, polyploidisation and multinucleation are characterised by the re-entry of a CM into the cell cycle, where it undergoes DNA synthesis during the S phase and nuclear division during the anaphase of M phase but does not complete cytokinesis. However, most current studies of cardiac regeneration applied the numbers or percentages of pH3+, aurora B+, EdU+/BrdU+ and PCNA+ CM to evaluate the regenerative condition of an injured heart, which is difficult to prove CM proliferation. Histone H3 phosphorylation is required to initiate DNA condensation^49,50^, which occurs during the S/G2 phase of cell cycle. Aurora B kinase directs thee localisation of centralspindlin to the central spindle^25^ during the anaphase of M phase and can thus be considered a marker of karyokinesis. Previous studies have found that EdU/BrdU incorporation experiments only account for DNA synthesis^51^, whilst PCNA (similar to pH3, EdU and BrdU) stains CMs that are entering cell cycle^52^. In other words, none of these markers provide a suitable indication of CM proliferation. By contrast, we investigated the cytokinesis marker, mklp2, is required for cell abscission, the final step in cytokinesis^17–20^. Therefore, despite its infrequent use in previous studies, mklp2 may be a more powerful marker of cardiac regeneration and CM cytokinesis. The correct localisation of anillin represents a key event in the cytokinetic process^25^ because it affects the function of the actomyosin constriction ring, which can be used to evaluate cytokinesis. In addition, we evaluated ECT2 as another important marker of cytokinesis required for the correct localisation of anillin and structural integration of the actomyosin constriction ring^24^. Compared with diploid CMs, polyploid CMs regenerate substantial numbers of new CMs via cytokinesis, rather than cell cycle re-entry. This process is theoretically more easily achieved would thus allow the production of larger numbers of polyploid CM in the contexts of some heart diseases, such as cyanotic CHD. Therefore, the existence of polyploid and multinucleated CMs suggests that cardiac regeneration research should also focus on cytokinesis, rather than merely the G1/S phase of cell cycle or anaphase of mitosis.

Most previous studies elucidated the mechanisms of polyploidisation and multinucleation. By contrast, few addressed the biological significance of the cytokinesis of polyploid and multinucleated CMs. These latter studies found observed reduced or similar proliferative abilities in binucleated CMs relative to mononucleated CMs^11,12^. Despite the observation of binucleated CM cytokinesis, however, the ploidy of binucleated CM and mechanism by which the limitation of CM cytokinesis was bypassed remained unclear. As noted previously, defective anillin localisation cause a defect in actomyosin ring function and, consequently, cytokinesis failure^25^. Moreover, MgcRacGAP as a cytoskeleton regulator is also dislocated in the actomyosin ring in our experiments. We found that β-catenin could induce the expression of ECT2, another vital regulator of cytokinesis that further controls the localisation of anillin and MgcRacGAP, and thus promotes cytokinesis (Supplemental Fig. 9).

The Wnt/β-catenin signalling pathway is a main driving force during cardiogenesis^53^. However, β-catenin plays dual roles in cardiac development and regeneration^54–58^. During development, active β-catenin is required for the differentiation of pre-cardiac mesoderm^59^, whereas the differentiation of cardiac precursor cells requires the inhibition of β-catenin^58^. In the adult heart, β-catenin promotes cardiac fibroblast proliferation to accelerate fibrosis^60^ and is required for cardiac stem cell proliferation^55^. Although several studies reported the beneficial effect of β-catenin on CM entry into the cell cycle^61,62^, we did not find a published study of its role in CM (particularly polyploid CM) cytokinesis. Our results from this study have therefore expanded the existing knowledge of various polyploid multinucleated CM subsets and the role of β-catenin in promoting CM cytokinesis. However, these results represent merely the tip of the iceberg, and further investigations are needed to clarify the mechanism by which CM cytokinesis is limited.

## Materials and Methods

### Animals

C57BL/6J mice were housed and handled at Xinqiao Hospital according to the institutional guidelines and in compliance with the Guide for the Care and Use of Laboratory Animals^63^. All experimental procedures were approved by the Animal Welfare Committee of Xinqiao Hospital. All mice were housed in an environment with controlled light cycles (12 h light/12 h dark), temperature, and humidity, and food and water provided ad libitum. All surgeries and subsequent analyses were performed in a blinded fashion.

### Hypoxic animal model

A hypoxia workstation (Baker Ruskinn InvivO2 1000; Ruskinn Technology, Ltd., Bridgend, UK) was used to establish a hypoxic animal model by exposing mice to an environment of 10% O_2_ for 4 weeks^64^. Normoxic mice were housed in an environment of 21% O_2_ and otherwise identical conditions for 4 weeks.

### Drug injection

The GSK-3β inhibitor CHIR99021 (Sigma, St. Louis, MO) was used as a β-catenin activator^62^. Both CHIR99021 and the β-catenin inhibitor IWR-1 (Sigma) were dissolved in dimethyl sulfoxide (DMSO) to generate stock solutions, which were then diluted in phosphate-buffered saline solution (PBS) before use. All the mice exposed for 3 weeks hypoxia were weighed daily and divided into three groups (h3w + c1w, h3w + i1w, h3w + d1w) that received subcutaneous injections of 2 mg/kg CHIR99021, 1.75 mg/kg IWR-1 and an equal volume of DMSO, respectively, each day for 4^th^ week hypoxia period.

### Evaluation of haemodynamics

After a 4-week exposure to hypoxia, the mice were subjected to cardiac catheterisation and haemodynamic analysis, as previously described^65^. Briefly, each mouse was anaesthetised via inhaled isoflurane, and the chest was opened via a median sternotomy. A 1.4-F Millar microtip catheter transducer (SPR-839, Millar Instruments, Houston, TX) was inserted to the left ventricular cavity to allow continuous recording of pressure-volume signals. PVAN software (Millar Instruments) was used to analyse the pressure–volume loops according to the manufacturer’s instructions.

### Immunofluorescent staining

Frozen human and mouse tissues were sectioned into 6-μm-thick slices and fixed in 4% paraformaldehyde (PFA) for 10 min. Cultured cells were fixed in 4% PFA for 20 min. The fixed heart sections and cultured cell samples were then permeabilised with 0.1% Triton X-100, blocked with 10% goat serum (AR0009; Boster Systems, Inc., Pleasanton, CA) and incubated overnight with primary antibodies at 4 °C. After three washes with PBS, the samples were incubated with secondary antibodies for 1 h at 37 °C and stained with the nuclear marker 4′,6-diamidino-2-phenylindole dihydrochloride (DAPI) for 10 minutes. The stained samples were visualized using a Leica SP5 confocal microscope (Leica Microsystems, Inc., Buffalo Grove, IL) and quantified using ImageJ 2.1 software (National Institutes of Health, Bethesda, MD). The following primary antibodies were used: anti-cardiac troponin T (TnT; cat. no. ab8295; 1:500; Abcam, Cambridge, UK); anti-phospho histone H3 Ser10 (pH3; cat. no. 06–570; 1:200; EMD Millipore, Billerica, MA); anti-Aurora B (cat. no. ab2254; 1:200; Abcam, Cambridge, UK); anti-kif20a (sc-374508; 1:100; Santa Cruz Biotechnology, Dallas, TX); anti-Ki67 (ab15580; 1:200; Abcam, Cambridge, UK); anti-β-catenin (cat. no. 8480; 1:200; Cell Signaling Technology, Danvers, MA) and anti-wheat germ agglutinin (WGA) conjugated to Alexa Fluor 488 (cat. no. W11261; 1:200; Invitrogen/Thermo Fisher Scientific, Inc., Carlsbad, CA).

### CM isolation of adult mice hearts

The harvested hearts were washed with cold PBS and minced and incubated with 1 mg/ml collagenase II (Sigma-Aldrich; Merck KGaA, Darmstadt, Germany) and 0.15 mg/ml DNase I (cat. no. 10104159001; Roche Diagnostics GmbH, Mannheim, Germany) for 30 min at 37 °C. The supernatants were collected, passed through a 40-mm filter, centrifuged at 1000 rpm for 5 min and resuspended in PBS containing 2% foetal calf serum (FCS; Gibco/Thermo Fisher Scientific, Inc., Waltham, MA). CMs were counted using a haemocytometer.

### Flow cytometry and sorting

A single-cell suspension was produced by digesting cultured cells with 2.5 mg/ml trypsin (Amresco Life Science/VWR, Solon, OH). Suspensions of tissues and cultured cells were incubated with anti-cardiac troponin T-FITC (MACS; Miltenyi Biotec GmbH, Bergisch Gladbach, Germany), pyridine iodide (PI) and 0.1% Triton X-100 for 30 min at 4 °C. The cells were then washed twice in PBS containing 2% FCS. The samples were analysed on a flow cytometer (AccuriC6; BD Biosciences, Franklin Lakes, NJ), and FlowJo 10.0 software (Tree Star, Inc., Ashland, OR) was used for data analysis (Supplemental Fig. 12).

For sorting, cultured cells were digested with 0.1% trypsin to yield a single-cell suspension and incubated in 5 μg/ml Hoechst (Beyotime, Shanghai, China) for 10 min at 37 °C. After washing twice, the cells were resuspended in PBS containing 2% FCS. Tetraploid CMs were sorted using a FACSAria iii (BD; Becton, Dickinson and Company, NJ).

### Detection of ploidy and nuclear number

Nuclear ploidy was determined using flow cytometry. For nuclear ploidy, the cell membranes were lysed with a hypotonic lysis buffer (0.25 M glucose, 3 mm NaCl) for 30 min, followed by centrifugation at 1500rpm for 15min. The nuclear fraction was then resuspended in a solution containing PI and a PCM1-specific antibody (Santa Cruz Biotechnology, Dallas, TX) for 30 min, washed and incubated with an Alexa Fluor 488-conjugaged secondary antibody for 30 min. Nuclear ploidy was detected using an AccuriC6 flow cytometer.

The numbers of nuclei were detected in isolated adult CMs that were mounted on slides and stained with Hoechst and TnT antibody to detect the nuclei and cytoplasm, respectively, using the standard immunofluorescence protocol described above. The treated slides were examined by fluorescence microscope cytometry to yield observer-independent quantitative analyses of the nuclear number^66^.

To evaluate the distribution of polyploid multinucleated CMs *in vivo*, isolated CMs smears were subjected to immunofluorescence staining with Hoechst, a TnT antibody and WGA antibody to label the nuclei, cytoplasm and cell membrane, respectively. Confocal microscopy and ImageJ software were used to determine number of nuclei in each CM and the intensity of the DNA signal in each nucleus^67–70^ (Supplemental Fig. 11). In the human heart slice, only the nucleus surrounded completely and closely by Troponin T is identified as CM nucleus (Supplemental Fig. 8). Meanwhile, cellular ploidy was determined by the sum of every nuclear ploidy in one CM.

Finally, the ploidy condition of polyploid multinucleated CMs has been expressed as N*P c, where N is the nuclear number, P is the ploidy of each nucleus and c represents the haploid DNA content.

### Determination of cell death

Cell death was determined by TUNEL assay. Frozen sections and fixed cultured cells were stained with TnT antibodies and incubated with a TUNEL stain (*In Situ* Cell Death Detection Kit, POD, Roche Diagnostics GmbH, Mannheim, Germany). The sections were then co-stained with DAPI and visualised using a Leica SP5 confocal microscopy system. The images were analysed using ImageJ 2.1 software.

### WGA staining and quantify cell size

The cell size was detected by WGA staining using the standard immunofluorescence protocol described above. To quantify the cell size, each independent sample with 3 different fields and positions, each from left and right ventricles, and septum were captured at 400x magnification. ImageJ 2.1 software was used to quantify the size of each cell.

### Capillary density and size

Tissues were stained with anti-PECAM (CD31) primary antibody (1:50; Abcam, Cambridge, UK) and anti-cTnT antibody at 4 °C overnight, followed by a secondary antibody and anti-WGA staining. Capillary density and size were analyzed by ImageJ 2.1 software.

### Cell proliferation assay

Cell proliferation was assessed using a Cell Counting Kit (CCK8, CW608, Dojindo, Kumamoto, Japan) according to the manufacturer’s instructions. Briefly, CMs were seeded into a 96-well plate at a density of 1 × 10^5^ cells/ml. After hypoxia for 48 hours, ten microliters of CCK8 solution and 100 μl of Dulbecco’s modified Eagle’s medium (DMEM) containing 10% FCS were sequentially added to each well, and the absorbance was read at 450 nm.

### ChIP-qPCR

Chromatin immunoprecipitation (ChIP) was performed using the ChIP Assay Kit (Beyotime, China) according to the manufacturer’s protocol. Briefly, cultured cells and fresh tissue were fixed in 1% formaldehyde for 15 min at room temperature and neutralized with glycine solution (10×) for 5 min. The cells were resuspended using SDS lysis buffer with 100 mm PMSF, and then sonicated on ice to break the genome into 200–400 bp in size. An equal amount of chromatin was incubated overnight at 4 °C with β-catenin antibody (Santa, 1:200) and normal Rabbit IgG, and then added the Protein A + G Agarose beads (Beyotime, China) for further 1 h incubation. Sequentially the beads were washed, and the precipitate was eluted in ChIP elution buffer. The DNA in the precipitation was extracted using a standard phenol–chloroform method. After purification, the obtained DNA was amplified by quantitative PCR using primers flanking the β-catenin-regulated sites (Supplemental Table S3).

### Quantitative real-time RT-PCR and western blot analyses

Quantitative real-time PCR and western blotting analyses were performed as previously described^71^. Briefly, total RNA was extracted from myocardial samples and cultured CMs using TRIzol reagent (Invitrogen), and reverse-transcribed into cDNA using oligo (dT) primers and the PrimeScript RT reagent Kit (Takara Bio, Shiga, Japan). The PCR products were quantified using the SYBR Green PCR Master Mix (Takara) and normalized to β-actin. The real-time PCR primers used in this study are shown in Supplemental Table S2.

For western blotting, cardiac tissues and CMs were lysed in RIPA buffer supplemented with a protease inhibitor (0.5 mm PMSF; Beyotime Institute of Biotechnology) and phosphatase inhibitor (1 tablet/10 ml; Roche Diagnostics GmbH). The nuclear and cytoplasmic protein were extracted from cardiac tissue and cultured CM by using nuclear and cytoplasmic protein extraction kit (Beyotime) according to the manufacturer’s instructions. The protein concentrations of the samples were measured using the Bradford method, and 20 µg of each protein extract were separated by SDS-PAGE (Invitrogen, NP0301BOX) and transferred to a polyvinylidene difluoride (PVDF) membrane (Roche Diagnostics GmbH). The membrane was blocked with 5% BSA for 90 min at room temperature, incubated overnight with primary antibodies at 4 °C, treated with a peroxidase-conjugated secondary antibody for 1 h at 37 °C and detected using an enhanced chemiluminescence kit (Beyotime Institute of Biotechnology). Signals on the membranes were quantified via densitometry using ImageQuant TL software (General Electric, Co., Fairfield, CT). The following primary antibodies were used: anti-HIF-1α (abcam), anti-β-catenin (CST), anti-Laminin B1 (abcam) and anti-α-actin (abcam).

### Co-immunoprecipitation

The cultured cells were lysed with 1 ml of IP lysis buffer (Beyotime) with 1% PMSF for 30 min at 4 °C. After the lysates were centrifuged at 12,000 × g for 30 min at 4 °C, the supernatant was collected, added into 5 mg of antibodies, followed by incubation with Protein A/G agarose beads (Beyotime) overnight at 4 °C. After washing with IP buffer for five times, the immunocomplexes were subjected to western blot for further analysis.

### NRCM culture, infection with recombinant adenoviral vectors and induction of hypoxia

Primary neonatal rat CM (NRCM) cultures were performed as described previously^72^. Briefly, 1- to 3-day-old Sprague–Dawley rats were killed by swift decapitation according to the Guide for the Care and Use of Laboratory Animals (United States National Institutes of Health). NRCMs were isolated from the hearts of these mice and seeded into 6-well culture plates at a density of 1 × 10^6^ cells/well in DMEM (Gibco; Thermo Fisher Scientific, Inc., Waltham, MA, USA) containing 10% FCS and 1% penicillin/streptomycin. Following a 48-h incubation, the culture medium was replaced with DMEM containing 0.1% FCS and AraC (0.1 mm) (Sigma), after which the NRCMs were infected with Adβ-catenin (Adβ-cat), AdGFP, Adshβ-catenin (Adshβ-cat) and AdshRNA (Cyagen Biosciences, Santa Clara, CA) at a multiplicity of infection of 10. Transgene expression was detected in 95% to 100% of the cells. Next, the NRCMs were exposed to a 1% O_2_ environment in a CO_2_ incubator (Thermo Fisher Scientific) for 48 hours to induce a hypoxia cell model. Control cells were cultured under normoxic conditions.

### Determination of spontaneous contraction in proliferated CMs

Before exposed to 1% O_2_ environment, the NRCMs were infected with Premo FUCCI Cell Cycle Sensor (Invitrogen; Thermo Fisher Scientific, Inc., Waltham, MA, USA) according to the manufacturer’s protocol. After hypoxia for 48 hours, GFP+ or RFP+ CMs were regarded as proliferated CMs and GFP-RFP- CMs were in G0 phase as a control group. The rate of spontaneous contraction was determined by confocal live cell imaging (Leica).

### Human heart samples

All procedures involving human tissue samples were performed according to the principles outlined in the Declaration of Helsinki and approved by the Human Ethics Committee of Xinqiao Hospital (Chongqing, China). Informed consent was obtained from all subjects whose samples were included in this study. Samples were taken from the stenotic right ventricular outflow tracts of patients with cyanotic and acyanotic congenital heart disease (CHD). A portion of each tissue was processed for flow cytometric ploidy detection, and the remainder was stored immediately at −80 °C for subsequent experiments. The study included 10 acyanotic and 10 cyanotic patients who were treated at Xinqiao Hospital (Chongqing, China) from June 2014 to October 2015. The clinical characteristics of the patients are presented in Supplemental Table S1.

### Data processing and gene expression profile mining

Three datasets of diploid and polyploid CMs were downloaded from the NCBI GEO (http://www.ncbi.nlm.nih.gov/geo/) and ArrayExpress (https://www.ebi.ac.uk/arrayexpress/) databases: GSE64403, GSE95755 and GSE95762. Sequence alignment and assembly were performed as described previously^73,74^. The criteria for differential mRNA expression included a fold change greater than 1.5 or less than 0.67 and a P value of less than 0.05. A Venn plot was generated using the Bioinformatics and Evolutionary Genomics web tool (http://bioinformatics.psb.ugent.be/webtools/Venn/), and heat maps were visualized using HemI 1.0^75^. The DAVID website (https://david.ncifcrf.gov/home.jsp) was used to conduct a gene ontology (GO) analysis, and the KEGG PATHWAY Database (http://www.genome.jp/kegg/pathway.html) was applied to the pathway analysis.

### Statistical analysis

All statistical analyses were performed using GraphPad Prism 6.0 software (GraphPad Software, Inc., La Jolla, CA). The data are presented as the means ± standard deviation. Comparisons between two groups were performed using an unpaired, two-tailed Student’s *t*-test. Differences among groups were evaluated using an analysis of variance, followed by a post hoc Tukey’s test. A P value of less than 0.05 was considered to indicate a statistically significant difference.

## Supplementary information

Supplementary information
