# Supplementary material for: Re-enforcing hypoxia-induced polyploid cardiomyocytes enter cytokinesis through activation of β-catenin
Source: Sci Rep. 2019 Nov 28;9:17865. doi: 10.1038/s41598-019-54334-4 (PMC6883062; doi:10.1038/s41598-019-54334-4)
Supplement: Supplementary file 1 — Supplementary information [file 41598_2019_54334_MOESM1_ESM.pdf]

Re-enforcing hypoxia-induced polyploid cardiomyocytes enter cytokinesis through activation of β-catenin

Yun-Han Jiang [1], Yu Zhu [1], Sai Chen [1], Hai-Long Wang [1], Yang Zhou [2], Fu-Qin Tang [1], Zhao Jian [1], Ying-Bin Xiao [1]*

1. Department of Cardiovascular Surgery, Xinqiao Hospital, Army Medical University, Chongqing, 400037, P.R. China; 2. Department of Cardiothoracic Surgery, the People's Hospital of Leshan, Leshan, Sichuan Province, 614000, P.R. China

* Correspondence and requests for materials should be addressed to Y.B.X. (email: xiaoyb@tmmu.edu.cn)

Running title: Cytokinesis of polyploid cardiomyocytes

Supplemental Figure S1. Active β-catenin promotes tetraploid CM cytokinesis by increasing the cytokinesis factors ECT2 expression.

(a) Location of the predicted β-catenin-regulated motif of *ECT2 gene*.
(b) The ChIP-qPCR result of two predicted motifs in CHIR9901-treated mice heart.
(c) The ChIP-qPCR result of two predicted motifs in CHIR9901-treated NRCM.
(d) Representative co-immunofluorescence with anti-ECT2, anti-Anillin and anti-cTnT antibodies. Scale bars, 5μm.
(e) The percent of ECT2-positive and Anillin-positive NRCMs in both normoxic and hypoxic group (n=20 samples each).
(f) The percent of ECT2-positive and Anillin-positive NRCMs among three groups (n=10 samples each).
(g) Representative co-immunofluorescence with anti-ECT2, anti-MgcRacGAP and anti-cTnT antibodies. Scale bars, 5μm.
(h) The percent of ECT2-positive and MgcRacGAP-positive NRCMs in both normoxic and hypoxic group (n=40 samples each).
(i) The percent of ECT2-positive and MgcRacGAP-positive NRCMs across three groups (n=10 samples each).
(j) qPCR analysis of cell cycle and cytokinesis genes normalized to actin (n=3 each).
(k) Representative distribution ploidy of treated tetraploid NRCMs.
(l) Ploidy of treated tetraploid NRCMs (n=3 for DMSO group, n=5 for β-catenin overexpression group). Data is presented as mean ± s.d. * $P < 0.05$.

Supplemental Figure S2. Active β-catenin improve the cardiac function without increasing hypoxia-induced angiogenesis and inhibiting CM apoptosis.

(a) Immunofluorescence with anti-CD31 antibodies. Results are displayed as three representative images (upper, scale bars, 50μm), as three box plots of average capillary number per field (lower left, n=5 each), average capillary area per field (lower medium, n=5 each) and capillary number per cardiomyocytes (lower right, n=3 each), respectively.
(b) Co-immunostaining with TUNEL staining and anti-cTnT antibodies in NRCMs. Results are displayed as three representative images (upper, scale bars, 50μm) and as two box plots of TUNEL-positive cardiomyocyte nuclei (lower left, n=4 samples each) and TUNEL-positive cardiomyocytes (lower right, n=4 samples each).
(c) Co-immunostaining with TUNEL staining, WGA staining and anti-cTnT antibodies in adult mice. Results are displayed as three representative images (upper, scale bars, 50μm) and as two plot charts of TUNEL-positive cardiomyocyte nuclei (lower left, n=3 each) and TUNEL-positive cardiomyocytes (lower right, n=3 each).
(d) Representative tracings of left-ventricular steady-state pressure-volume loops.
(e) Ejection fraction of left ventricular among three groups (n=10 each). Data is presented as mean ± s.d. * $P < 0.05$.

Supplemental Figure S3. Hypoxia may induce higher ploidy and nuclear number of cardiomyocytes in cyanotic congenital heart disease.

(a) The mean ploidy of cardiomyocyte nuclei between acyanotic and cyanotic group

via fluorescence intensity measurement (n=3 each).
(b) Distribution of cardiomyocyte ploidy (n=3 each).
(c) Correlation between mean nuclear number of cardiomyocytes and arterial oxygen saturation in congenital heart disease samples.
(d) Correlation between mean ploidy of cardiomyocyte nuclei and arterial oxygen saturation in both acyanotic and cyanotic groups together.
(e) Correlation between mean ploidy of cardiomyocytes and arterial oxygen saturation in both groups together. Data is presented as mean ± s.d. * $P < 0.05$.

Supplemental Figure S4. Ploidy and nuclear number of cardiomyocytes is higher in hypoxic heart.
(a) Representative distribution of ploidy of cardiomyocyte nuclei in both normoxic and hypoxic group.
(b) The percent of polyploid cardiomyocyte nuclei between normoxic and hypoxic group (n=4 each).
(c) The mean ploidy of cardiomyocyte nuclei via flow cytometry (n=4 each).
(d) Distribution of cardiomyocyte ploidy (n=3 each). Data is presented as mean ± s.d. * $P < 0.05$.

Supplemental Figure S5. Hypoxia induce higher ploidy and nuclear number of neonatal rat cardiomyocytes.
(a) The percent of polyploid neonatal rat cardiomyocyte nuclei between normoxic and hypoxic group (n=9 samples each).
(b) The mean ploidy of cardiomyocyte nuclei via fluorescence intensity measurement (n=9 samples each).
(c) Distribution of neonatal rat cardiomyocyte ploidy in both groups (n=18 samples each). Data is presented as mean ± s.d. * $P < 0.05$.

Supplemental Figure S6. Acive β-catenin increase the number of cardiomyocytes under hypoxia condition *in vitro* and *in vivo*.
(a) The OD value of CCK-8 in both normoxic and hypoxic group (n=3 each).
(b) The OD value of CCK-8 across three groups (n=3 each).
(c) Complete isolated cardiomyocyte count per heart in both normoxic and hypoxic group (n=4 each).
(d) Complete isolated cardiomyocyte count per heart across three groups (n=4 each).
(e) The ratio of heart weight to body weight (HW/BW) in both normoxic and hypoxic group (n=4 each).
(f) HW/BW across three groups (n=8 each).
(g) The ratio of heart weight to tibial length (HW/TL) in both normoxic and hypoxic group (n=4 each).
(h) HW/TL across three groups (n=8 each). Data is presented as mean ± s.d. * $P < 0.05$.
(i) The rate of spontaneous contraction (n=6 each). Data is presented as mean ± s.d. *$P < 0.05$.

Supplemental Figure S7. Acive β-catenin is unable to change the relative weight of lung.

(a) The ratio of lung weight to body weight (LW/BW) in both normoxic and hypoxic group (n=4 each).

(b) The ratio of lung weight to tibial length (LW/TL) in both normoxic and hypoxic group (n=4 each).

(c) LW/BW across three groups (n=8 each).

(d) LW/TL across three groups (n=8 each). Data is presented as mean ± s.d. * $P < 0.05$.

Supplemental Figure S8. Hypoxia induces angiogenesis, increases apoptosis and reduce cardiac function.

(a) Immunofluorescence with anti-CD31 antibodies. Results are displayed as two representative images (upper, scale bars, 50μm), as three box plots of average capillary number per field (lower left, n=9 each), average capillary area per field (lower medium, n=9 each) and capillary number per cardiomyocytes (lower right, n=3 each), respectively.

(b) Co-immunostaining with TUNEL staining and anti-cTnT antibodies in NRCMs. Results are displayed as two representative images (upper, scale bars, 50μm) and as two box plots of TUNEL-positive cardiomyocyte nuclei (lower left, n=4 samples each) and TUNEL-positive cardiomyocytes (lower right, n=4 samples each).

(c) Co-immunostaining with TUNEL staining, WGA staining and anti-cTnT antibodies in adult mice. Results are displayed as two representative images (upper, scale bars, 50μm) and as two plot charts of TUNEL-positive cardiomyocyte nuclei (lower left, n=3 each) and TUNEL-positive cardiomyocytes (lower right, n=3 each).

(d) Representative tracings of left-ventricular steady-state pressure-volume loops.

(e) Ejection fraction of left ventricular in both normoxic and hypoxic group (n=16 each). Data is presented as mean ± s.d. * $P < 0.05$.

Supplemental Figure S9. Anillin and ECT2 dislocated in hypoxic cardiomyocytes.

(a) The schematic diagram of dynamic location of ECT2, Anillin and actomyosin during cardiomyocyte cytokinesis under normoxic and hypoxic condition.

(b) Representative immunostaining image with anti-ECT2, anti-Anillin and anti-cTnT antibodies during anaphase. ECT2 and Anillin appear to accumulate at central spindle microtubules between two nuclei. Scale bars, 5μm.

(c) Representative image during kataphase. ECT2 and Anillin locate at actomyosin constriction ring. Scale bars, 5μm.

(d) Representative image during cytokinesis. ECT2 and Anillin located at intercellular bridge. Scale bars, 5μm.

(e) Representative image of cytokinesis failure. ECT2 and Anillin fail to accumulate at actomyosin ring. Scale bars, 5μm.

Supplemental Figure S10. The method for counting the cardiomyocyte nuclei.

Nucleus only surrounding by Troponin T closely is defined as cardiomyocyte nucleus (white arrowhead). The nucleus defined as nonmyocyte nucleus is not completely

surrounded by Troponin T (□ and ○) or not closely surrounded by Troponin T (#) or is partly (Δ) or completely (white arrow) surrounded by cell membrane.
(a) Scale bars, 10μm.
(b) Scale bars, 5μm.
(c) Scale bars, 5μm.
(d) Scale bars, 50μm.

Supplemental Figure S11. The method for measuring the ploidy and nuclear number of cardiomyocytes.
(a) The schematic flow diagram of detecting the distribution of multinucleated polyploid cardiomyocytes. The NRCM or the cardiomyocyte smear of animal model is stained by Hoechst, WGA and anti-cTnT antibodies, which marked the DNA, cytosol and membrane of myocyte, respectively.
(b) Immunofluorescence image is captured via confocal laser scanning microscope (scale bars, 50μm).
(c) According to the inclusion and exclusion criteria, the cardiomyocyte nucleus is defined by the relationship among nucleus, Troponin T and membrane.
(d) The fluorescence intensity of Hoechst is detected to form the intensity curve.
(e) The first curve peak is regard as diploid, and the second is regard as tetraploid, and so on. The intensity value of tetraploid need be about the two-fold one of diploid.
(f) Based on ploidy curve, the ploidy of each nucleus can be identified.
(g) The nuclear number of each cell can also be counted.
(h) Considering the nuclear number of each cell, the distribution of multinucleated polyploid cardiomyocyte can be obtained.

Supplemental Figure S12. The strategy of flow cytometry for the ploidy of isolated adult mice cardiomyocytes.
(a) According to the FITC-conjugated Troponin T (TnT) antibodies, the cardiomyocytes can be screened.
(b) According to the intensity of DNA dye (Pyridine iodide for analysis or Hoechst for cell sorting), different ploidy of cardiomyocytes can be identified after the removal of cell adhesions.
(c) Ploidy distribution of cardiomyocytes can be detected.

Supplemental Figure S12. The strategy of flow cytometry for the ploidy of isolated adult mice cardiomyocytes.
(a) According to the FITC-conjugated Troponin T (TnT) antibodies, the cardiomyocytes can be screened.
(b) According to the intensity of DNA dye (Pyridine iodide for analysis or Hoechst for cell sorting), different ploidy of cardiomyocytes can be identified after the removal of cell adhesions.
(c) Ploidy distribution of cardiomyocytes can be detected.

Supplemental Figure S13. Knocking down of ECT2 increases ploidy and nuclear number of neonatal rat cardiomyocytes with active β-catenin.

(a) Distribution of ploidy of NRCM. Results are displayed as a representative hypoxic cardiomyocytes (upper), as a bar chart of ploidy of cardiomyocytes (lower left, n=4 samples each) and as a box plot of mean ploidy of cardiomyocytes (lower right, n=4 samples each). (b) The nuclear number of NRCMs. Results are displayed as a representative hypoxic image (upper, scale bars, 50μm), as a bar chart of multinucleated cardiomyocytes (lower left, n=37 samples for normoxia, n=28 samples for hypoxia, >30 cardiomyocytes from 9 randomly chosen fields were counted for each individual sample) and as a chart for mean nuclear number (lower right). (c) Distribution of ploidy (left, n=12 samples each) and mean ploidy (right, n=12 samples each, >30 cardiomyocytes from 9 randomly chosen fields were counted for each individual sample) of NRCMs. (d) The percent of Ki67-positive cardiomyocyte nuclei (left, n=9 samples each, >30 cardiomyocytes from 9 randomly chosen fields were counted for each individual sample) and Ki67-positive cardiomyocytes (left, n=9 samples each, >30 cardiomyocytes from 9 randomly chosen fields were counted for each individual sample). (e) The percent of pH3-positive cardiomyocyte nuclei (left, n=8 samples each, >30 cardiomyocytes from 9 randomly chosen fields were counted for each individual sample) and pH3-positive cardiomyocytes (right, n=8 samples each, >30 cardiomyocytes from 9 randomly chosen fields were counted for each individual sample). (f) The percent of Aurora B-positive cardiomyocyte nuclei (left, n=8 samples each, >30 cardiomyocytes from 9 randomly chosen fields were counted for each individual sample) and Aurora B-positive cardiomyocytes (right, n=8 samples each, >30 cardiomyocytes from 9 randomly chosen fields were counted for each individual sample). (g) The percent of mklp2-positive cardiomyocyte nuclei (left, n=8 samples each, >30 cardiomyocytes from 9 randomly chosen fields were counted for each individual sample) and mklp2-positive cardiomyocytes (right, n=8 samples each, >30 cardiomyocytes from 9 randomly chosen fields were counted for each individual sample). Data is presented as mean ± s.d. * $P < 0.05$.

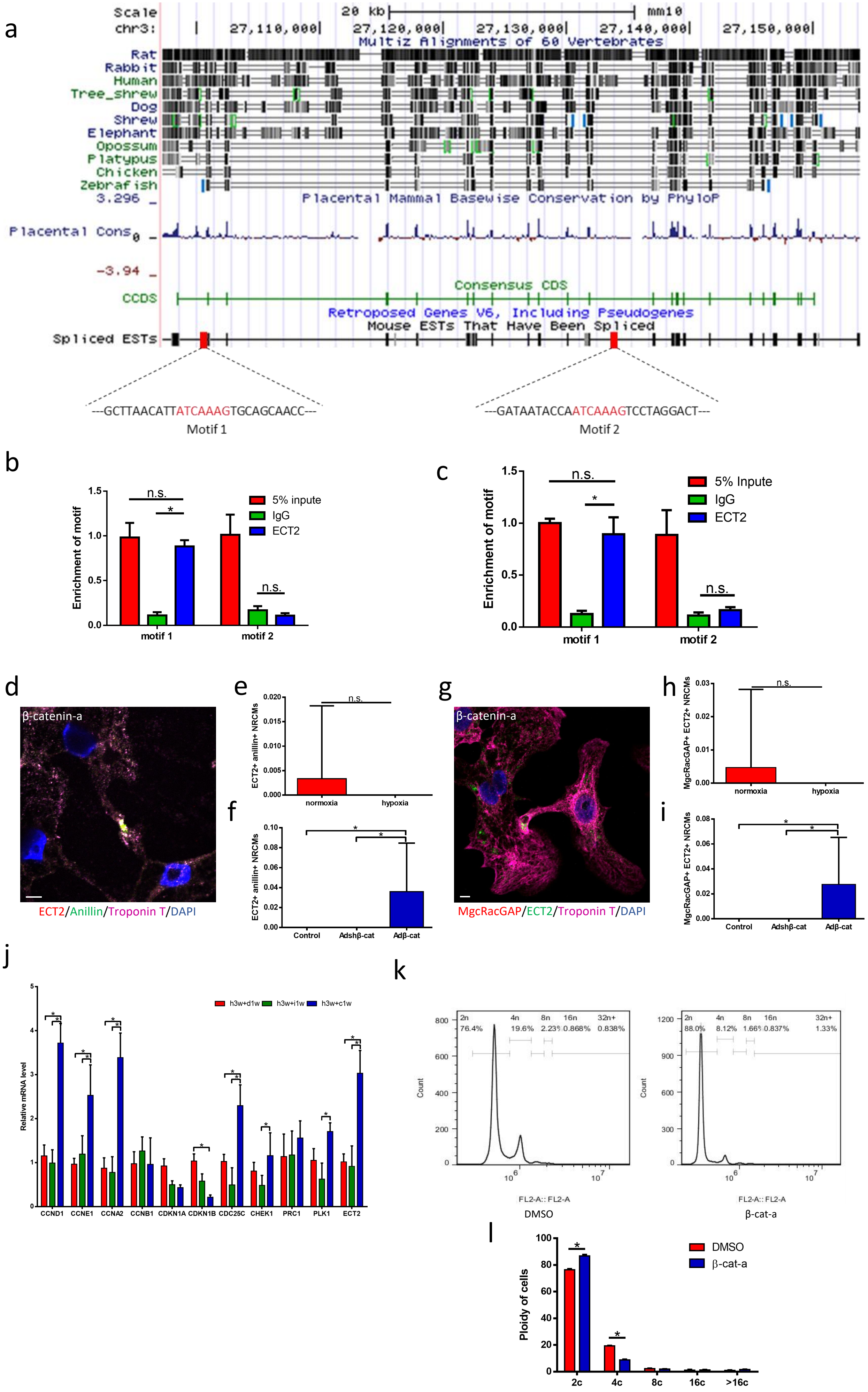
a
Scale
chr3:
20 kb
mm10
27,110,000|
27,120,000|
27,130,000|
27,140,000|
27,150,000|
Multiz Alignments of 60 Vertebrates
Rat
Rabbit
Human
Tree_shrew
Dog
Shrew
Elephant
Opossum
Platypus
Chicken
Zebrafish
3.296 _
Placental Mammal Basewise Conservation by PhyloP
Placental Cons 0 _
-3.94 _
Consensus CDS
CCDS
Retroposed Genes V6, Including Pseudogenes
Mouse ESTs That Have Been Spliced
Spliced ESTs
---GCTTAACATTATCAAAGTGCAGCAACC---
Motif 1
---GATAATACCAATCAAAGTCCTAGGACT---
Motif 2
b
Enrichment of motif
1.5
1.0
0.5
0.0
n.s.
*
n.s.
5% inpute
IgG
ECT2
motif 1
motif 2
c
Enrichment of motif
1.5
1.0
0.5
0.0
n.s.
*
n.s.
5% Inpute
IgG
ECT2
motif 1
motif 2
d
β-catenin-a
ECT2/Anillin/Troponin T/DAPI
e
ECT2+ anillin+ NRCMs
0.020
0.015
0.010
0.005
0.000
n.s.
normoxia
hypoxia
f
ECT2+ anillin+ NRCMs
0.10
0.08
0.06
0.04
0.02
0.00
*
*
Control
Adshβ-cat
Adβ-cat
g
β-catenin-a
MgcRacGAP/ECT2/Troponin T/DAPI
h
MgcRacGAP+ ECT2+ NRCMs
0.03
0.02
0.01
0.00
n.s.
normoxia
hypoxia
i
MgcRacGAP+ ECT2+ NRCMs
0.08
0.06
0.04
0.02
0.00
*
*
Control
Adshβ-cat
Adβ-cat
j
Relative mRNA level
5
4
3
2
1
0
h3w+d1w
h3w+i1w
h3w+c1w
CCND1
CCNE1
CCNA2
CCNB1
CDKN1A
CDKN1B
CDC25C
CHEK1
PRC1
PLK1
ECT2
k
2n
76.4%
4n
19.6%
8n
2.23%
16n
0.868%
32n+
0.838%
800
600
400
200
0
Count
10^6
10^7
FL2-A:: FL2-A
DMSO
2n
88.0%
4n
8.12%
8n
1.66%
16n
0.837%
32n+
1.33%
1200
900
600
300
0
Count
10^6
10^7
FL2-A:: FL2-A
β-cat-a
l
Ploidy of cells
100
80
60
40
20
0
*
*
DMSO
β-cat-a
2c
4c
8c
16c
>16c

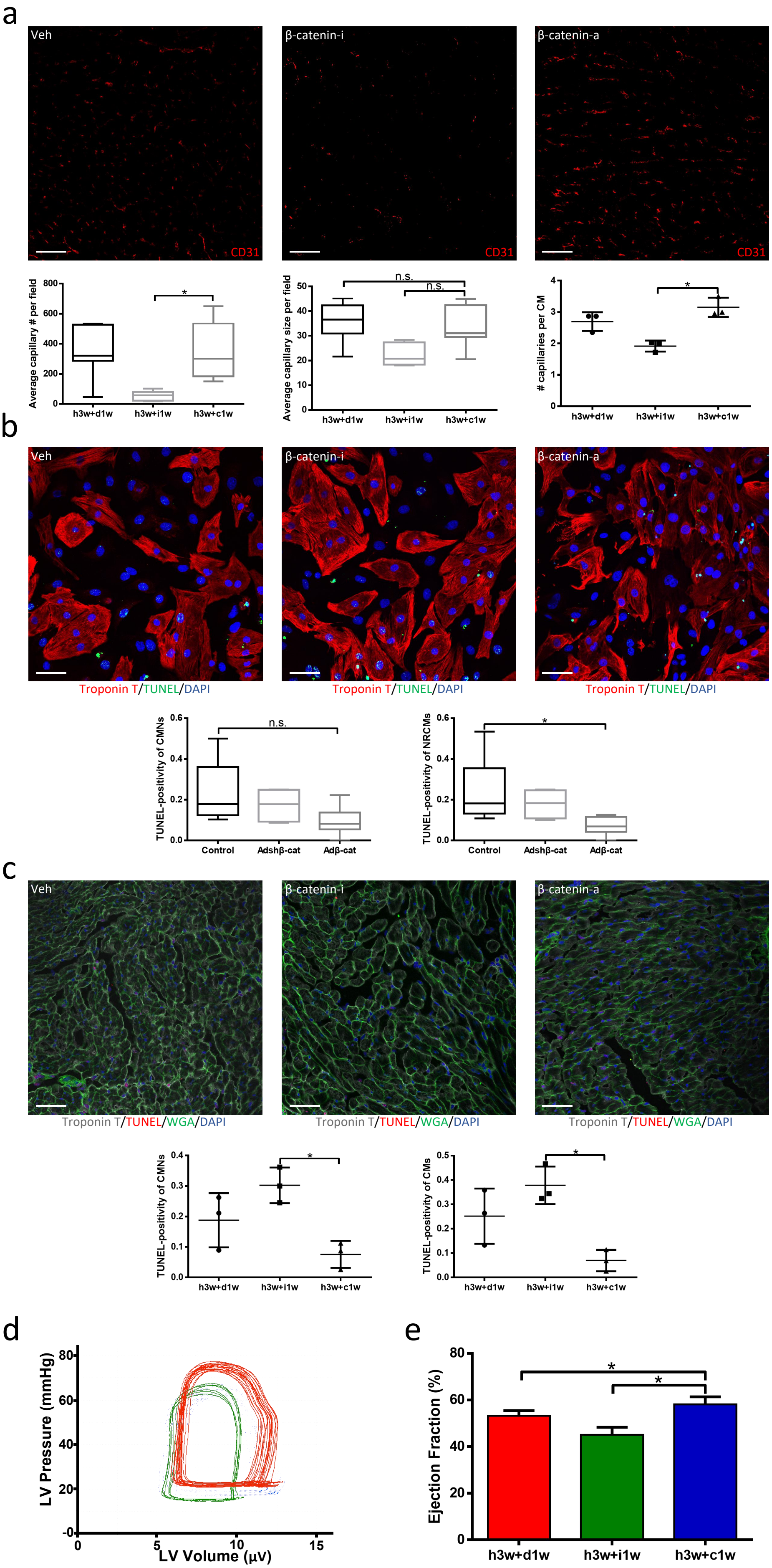

a
Veh
β-catenin-i
β-catenin-a
CD31
Average capillary # per field
h3w+d1w
h3w+i1w
h3w+c1w
Average capillary size per field
n.s.
capillaries per CM
b
Troponin T/TUNEL/DAPI
TUNEL-positivity of CMNs
TUNEL-positivity of NRCMs
Control
Adshβ-cat
Adβ-cat
c
Troponin T/TUNEL/WGA/DAPI
TUNEL-positivity of CMs
d
LV Pressure (mmHg)
LV Volume (μV)
e
Ejection Fraction (%)

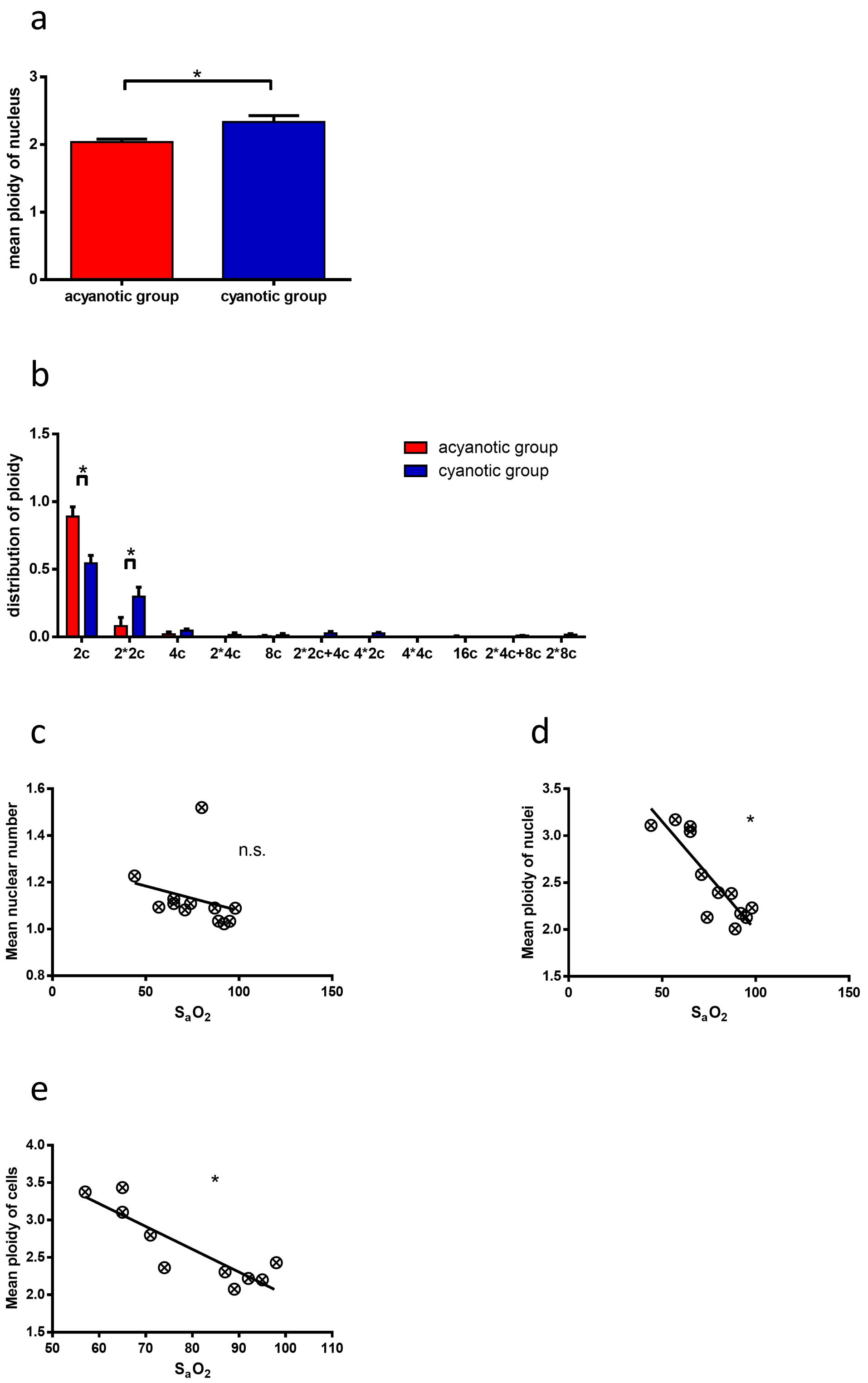
a
mean ploidy of nucleus
0
1
2
3
*
acyanotic group
cyanotic group
b
distribution of ploidy
0.0
0.5
1.0
1.5
*
*
acyanotic group
cyanotic group
2c
2*2c
4c
2*4c
8c
2*2c+4c
4*2c
4*4c
16c
2*4c+8c
2*8c
c
Mean nuclear number
0.8
1.0
1.2
1.4
1.6
n.s.
0
50
100
150
SaO2
d
Mean ploidy of nuclei
1.5
2.0
2.5
3.0
3.5
*
0
50
100
150
SaO2
e
Mean ploidy of cells
1.5
2.0
2.5
3.0
3.5
4.0
*
50
60
70
80
90
100
110
SaO2

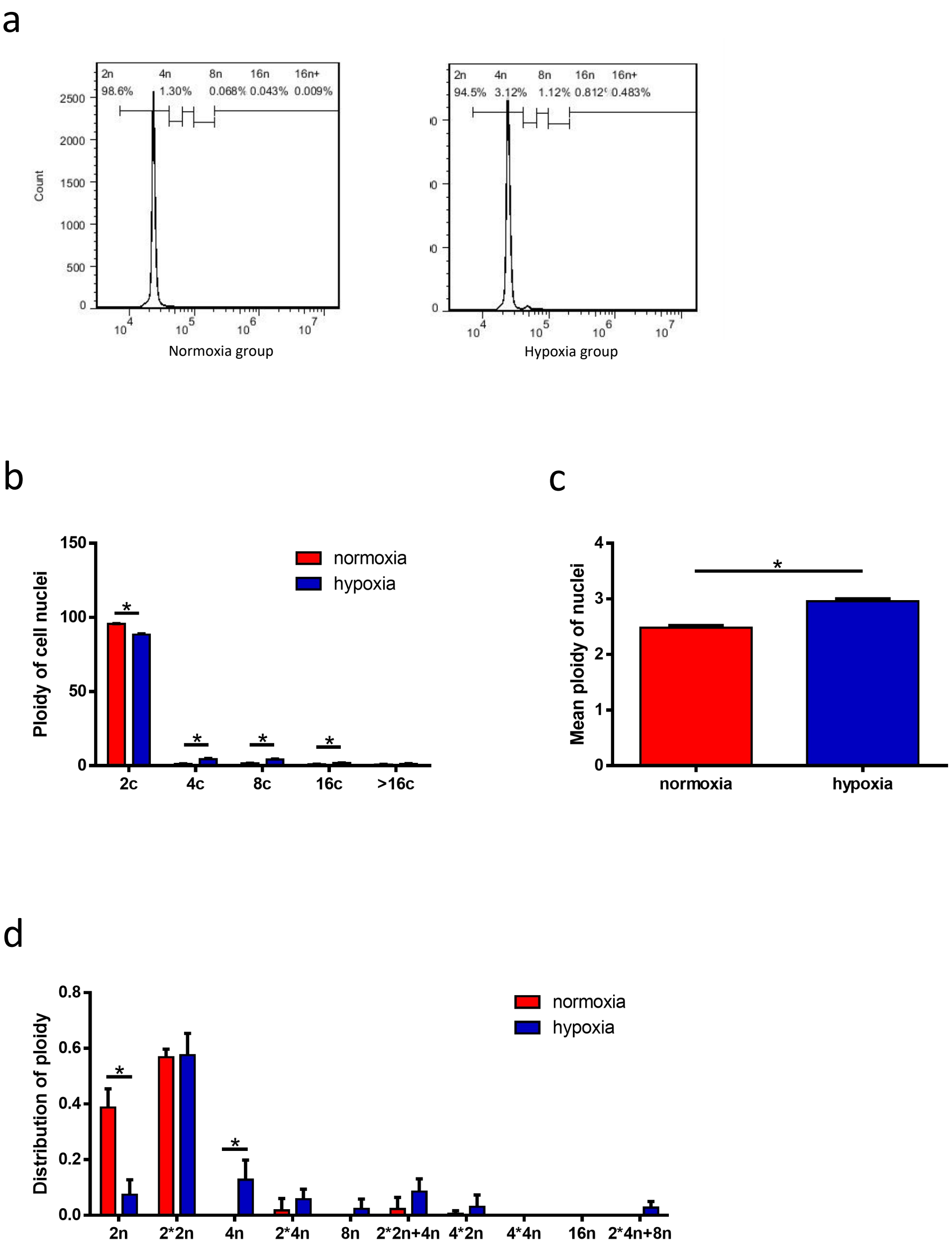

a
2n
98.6%
4n
1.30%
8n
0.068%
16n
0.043%
16n+
0.009%
Count
Normoxia group
2n
94.5%
4n
3.12%
8n
1.12%
16n
16n+
0.483%
Hypoxia group
b
Ploidy of cell nuclei
normoxia
hypoxia
2c
4c
8c
16c
>16c
c
Mean ploidy of nuclei
normoxia
hypoxia
d
Distribution of ploidy
normoxia
hypoxia
2n
2*2n
4n
2*4n
8n
2*2n+4n
4*2n
4*4n
16n
2*4n+8n

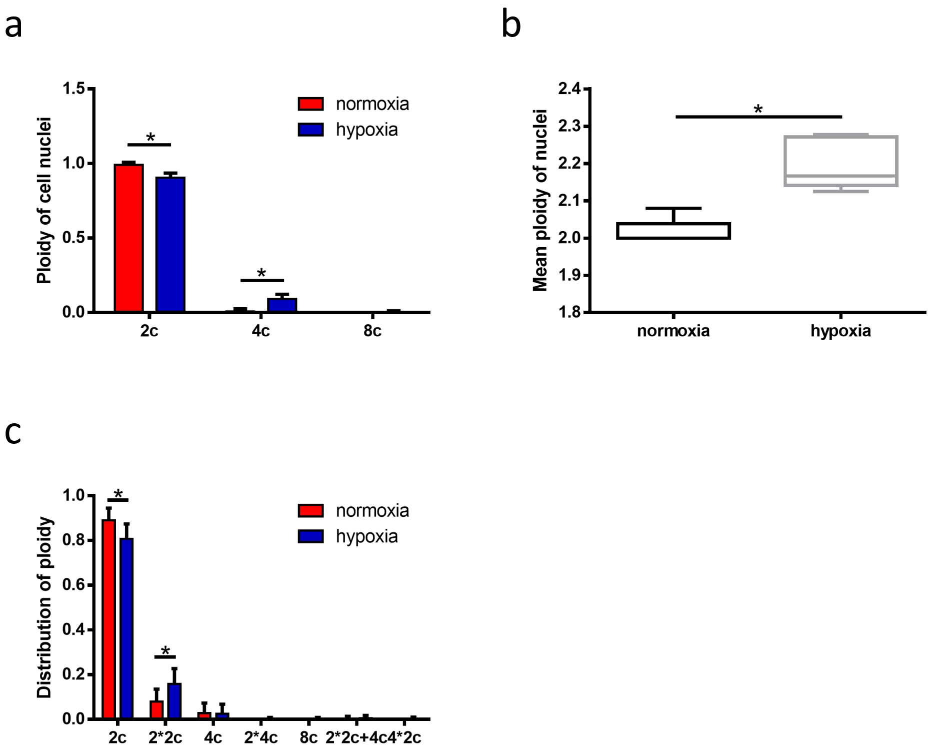
a
Ploidy of cell nuclei
1.5
1.0
0.5
0.0
2c
4c
8c
normoxia
hypoxia
*
*
b
Mean ploidy of nuclei
2.4
2.3
2.2
2.1
2.0
1.9
1.8
normoxia
hypoxia
*
c
Distribution of ploidy
1.0
0.8
0.6
0.4
0.2
0.0
2c
2*2c
4c
2*4c
8c
2*2c+4c
4*2c
normoxia
hypoxia
*
*

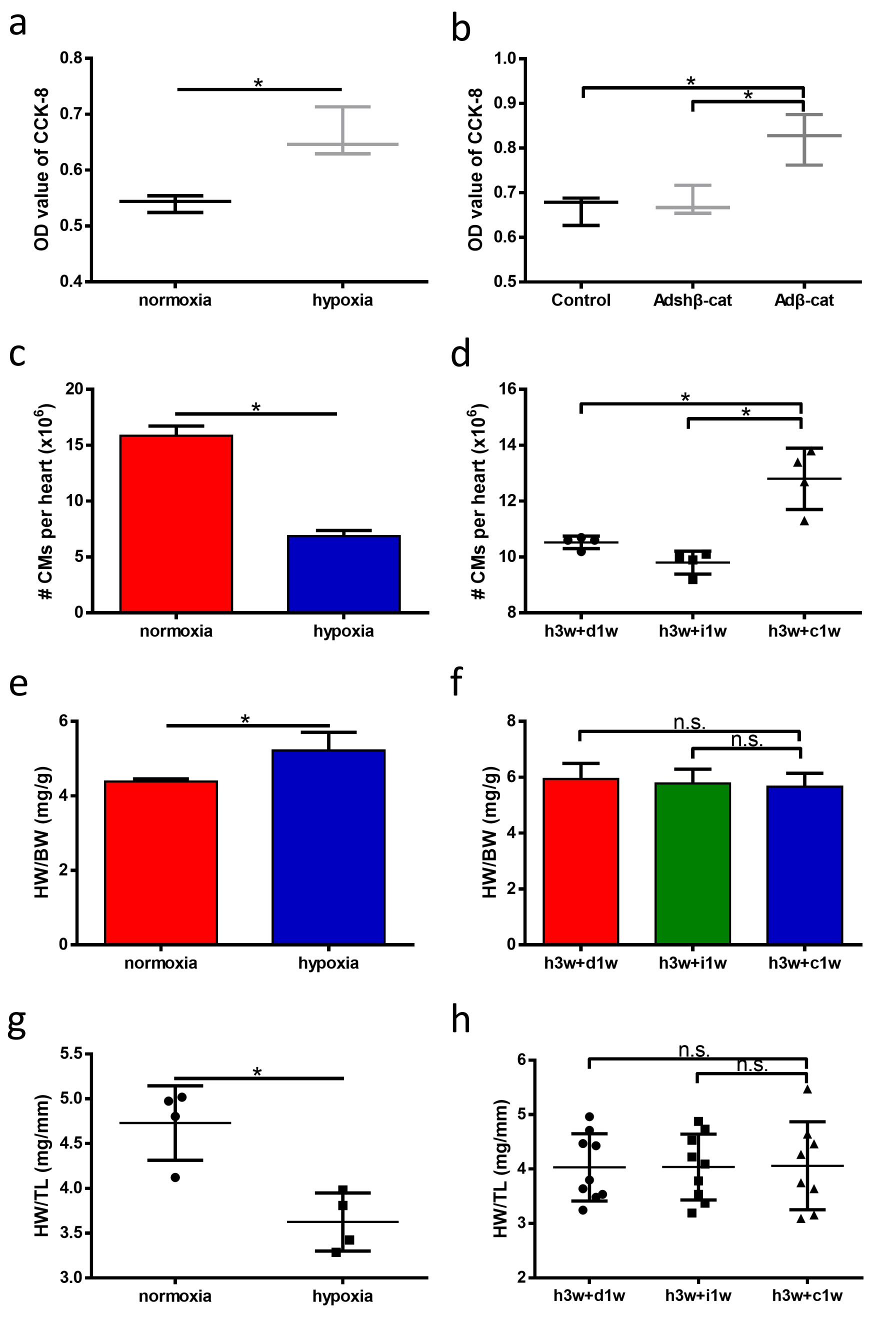
a
OD value of CCK-8
normoxia
hypoxia
b
OD value of CCK-8
Control
Adshβ-cat
Adβ-cat
c
CMs per heart (x10^6)
normoxia
hypoxia
d
CMs per heart (x10^6)
h3w+d1w
h3w+i1w
h3w+c1w
e
HW/BW (mg/g)
normoxia
hypoxia
f
HW/BW (mg/g)
n.s.
n.s.
h3w+d1w
h3w+i1w
h3w+c1w
g
HW/TL (mg/mm)
normoxia
hypoxia
h
HW/TL (mg/mm)
n.s.
n.s.
h3w+d1w
h3w+i1w
h3w+c1w

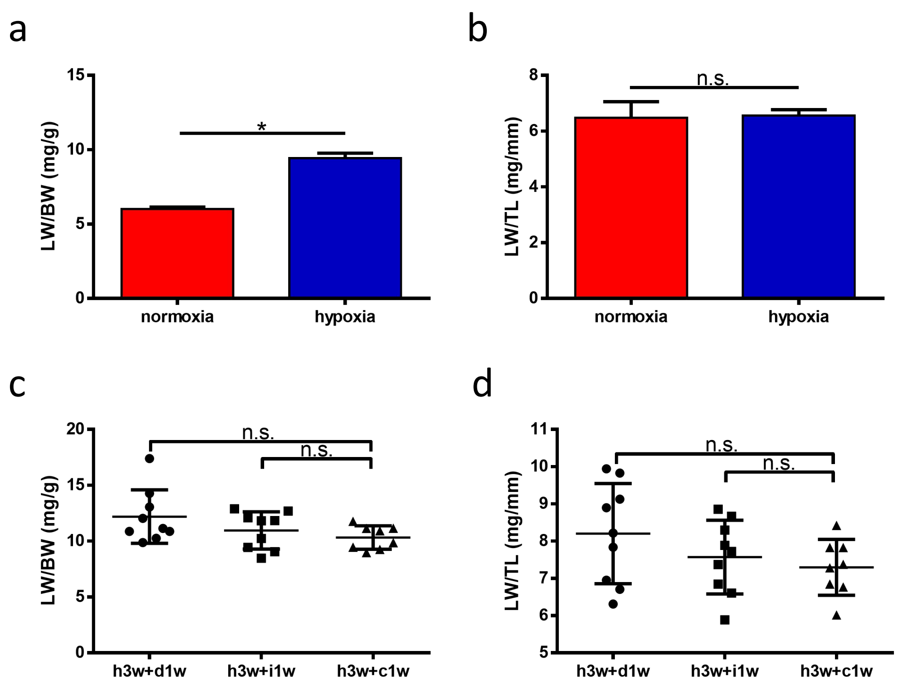
a
LW/BW (mg/g)
15
10
5
0
*
normoxia
hypoxia
b
LW/TL (mg/mm)
8
6
4
2
0
n.s.
normoxia
hypoxia
c
LW/BW (mg/g)
20
15
10
5
0
n.s.
n.s.
h3w+d1w
h3w+i1w
h3w+c1w
d
LW/TL (mg/mm)
11
10
9
8
7
6
5
n.s.
n.s.
h3w+d1w
h3w+i1w
h3w+c1w

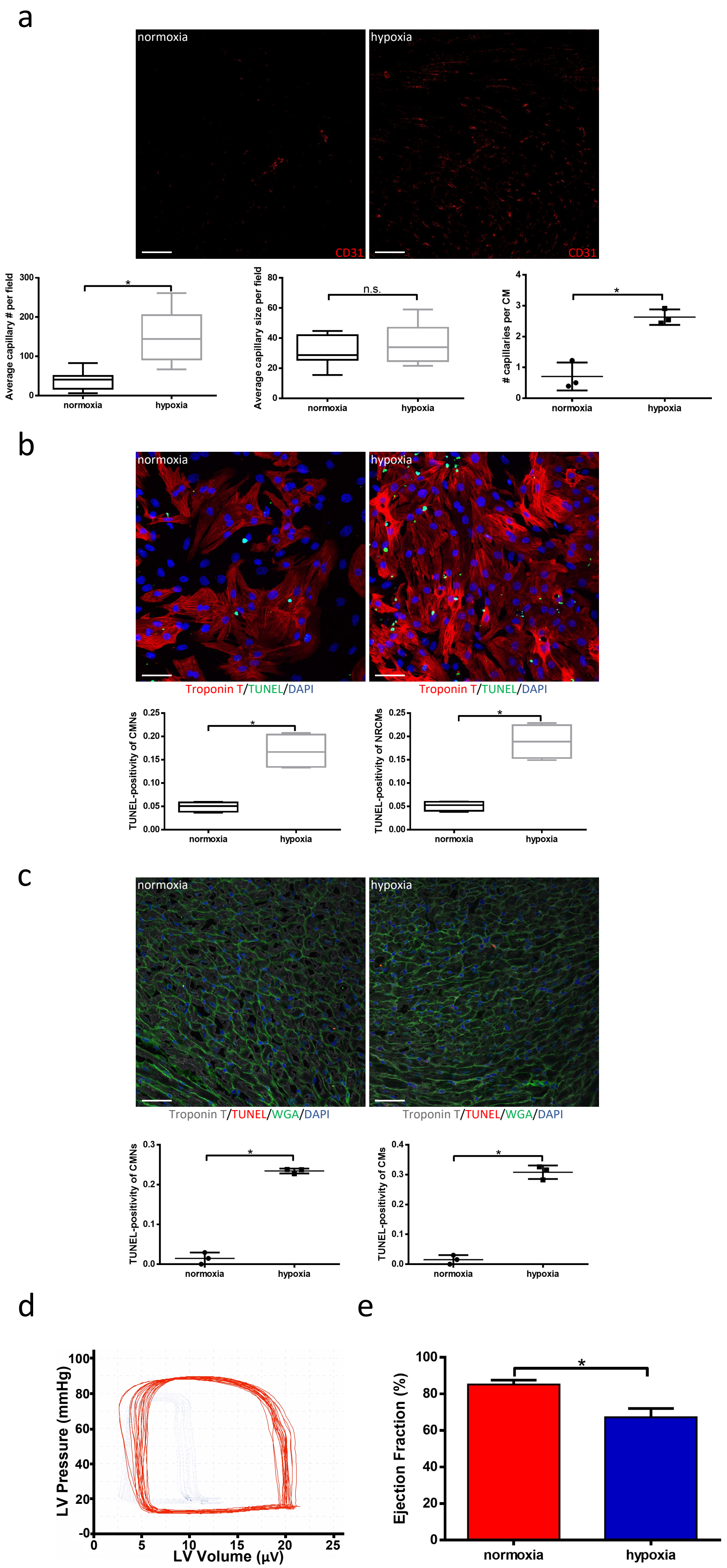
a
normoxia
hypoxia
CD31
CD31
Average capillary # per field
300
200
100
0
*
normoxia
hypoxia
Average capillary size per field
80
60
40
20
0
n.s.
normoxia
hypoxia
capillaries per CM
4
3
2
1
0
*
normoxia
hypoxia
b
normoxia
hypoxia
Troponin T/TUNEL/DAPI
Troponin T/TUNEL/DAPI
TUNEL-positivity of CMNs
0.25
0.20
0.15
0.10
0.05
0.00
*
normoxia
hypoxia
TUNEL-positivity of NRCMs
0.25
0.20
0.15
0.10
0.05
0.00
*
normoxia
hypoxia
c
normoxia
hypoxia
Troponin T/TUNEL/WGA/DAPI
Troponin T/TUNEL/WGA/DAPI
TUNEL-positivity of CMNs
0.3
0.2
0.1
0.0
*
normoxia
hypoxia
TUNEL-positivity of CMs
0.4
0.3
0.2
0.1
0.0
*
normoxia
hypoxia
d
LV Pressure (mmHg)
100
80
60
40
20
-0
0
5
10
15
20
25
LV Volume (μV)
e
Ejection Fraction (%)
100
80
60
40
20
0
*
normoxia
hypoxia

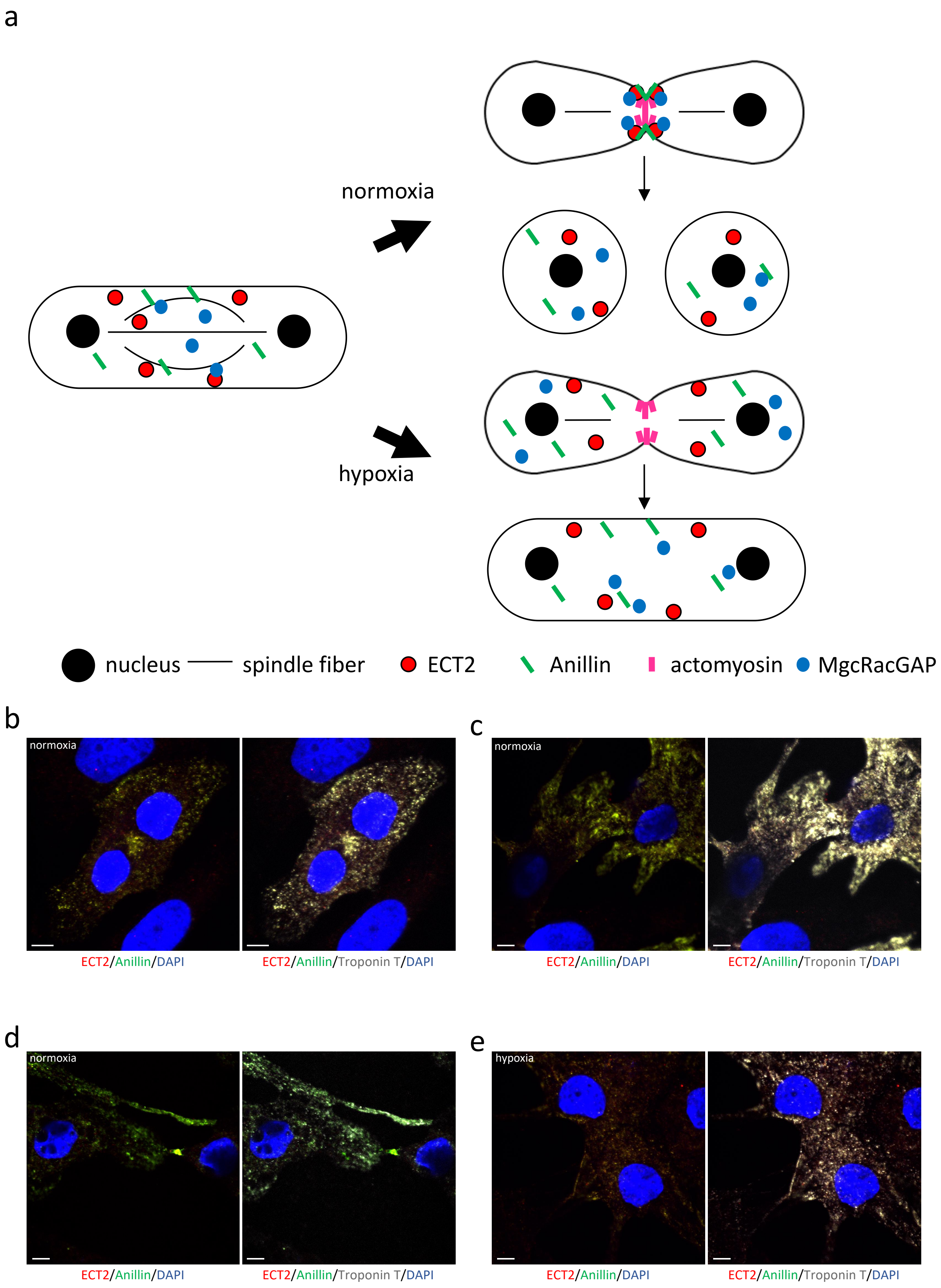
a
normoxia
hypoxia
nucleus
spindle fiber
ECT2
Anillin
actomyosin
MgcRacGAP
b
normoxia
ECT2/Anillin/DAPI
ECT2/Anillin/Troponin T/DAPI
c
normoxia
ECT2/Anillin/DAPI
ECT2/Anillin/Troponin T/DAPI
d
normoxia
ECT2/Anillin/DAPI
ECT2/Anillin/Troponin T/DAPI
e
hypoxia
ECT2/Anillin/DAPI
ECT2/Anillin/Troponin T/DAPI

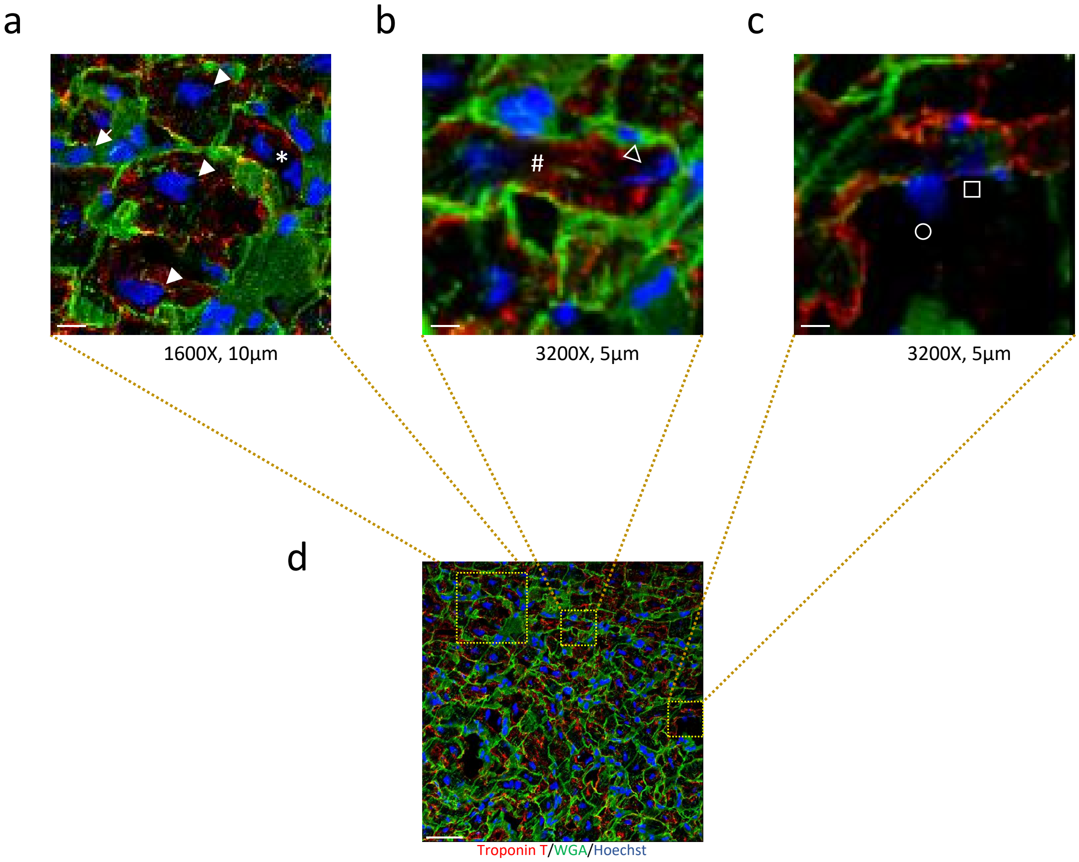
a
b
c
#
1600X, 10µm
3200X, 5µm
3200X, 5µm
d
Troponin T/WGA/Hoechst

a

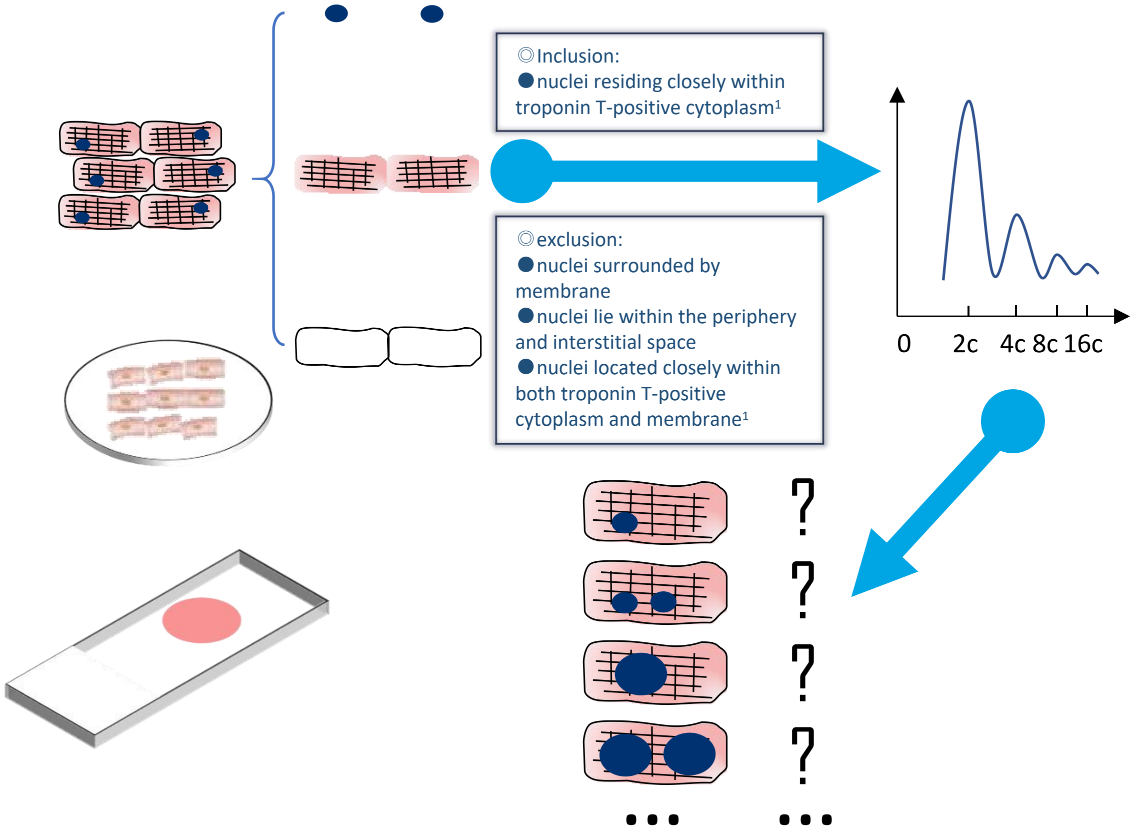

b c d

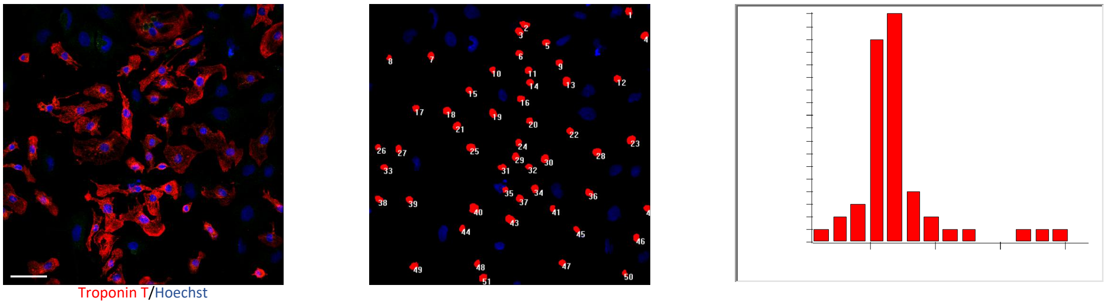

e

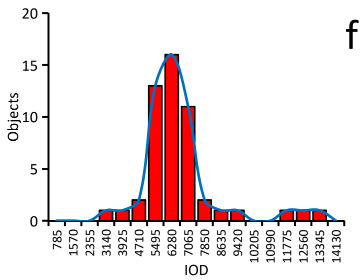

f

Ploidy of nuclei	Counts
2c	48
4c	3

g

Nuclear number	Counts
Nu=1	49
Nu=2	2

h

Distribution of ploidy	Counts
2c	46
2*2c	2
4c	3
2*4c	0
8c	0

1. Ang KL, Shenje LT, Reuter S, et al. Limitations of conventional approaches to identify myocyte nuclei in histologic sections of the heart. Am J Physiol Cell Physiol. 2010; 298(6):C1603-9.

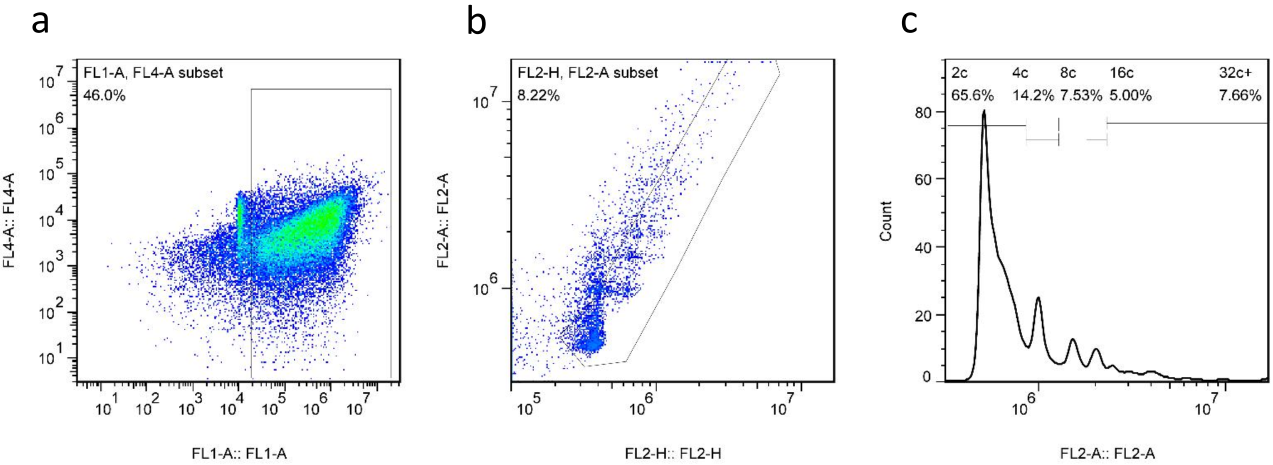

a
FL1-A, FL4-A subset
46.0%
FL4-A:: FL4-A
FL1-A:: FL1-A
b
FL2-H, FL2-A subset
8.22%
FL2-A:: FL2-A
FL2-H:: FL2-H
c
2c
65.6%
4c
14.2%
8c
7.53%
16c
5.00%
32c+
7.66%
Count
FL2-A:: FL2-A

Supplementary Table I

	Acyanotic group	Cyanotic group	P value
N	10	10	
Age	6.65±8.83	3.52±3.6	>0.05
Gender (male/female)	4/6	5/5	>0.05
Oxygen Saturation of Arterial Blood (%n)	94.12±4.72	68.57±11.37	<0.05

Supplementary Table II

Gene	FORWARD	REVERSE
CCND1	TGGATGCTGGAGGTCTGTGAGG	GCAGGCGGCTCTTCTTCAAGG
CHEK1	CTCAGAGGTTCTTCCACCAACTCATG	CACATCTTGTTCAGTAAGCGTTCACG
PRC1	AGTCAGTTGTCACTTCTCTGTGTTCTG	ATGCTGCCATTGAGGTCCAAGTTC
PLK1	TGCGGCAAGAGGAGGCTGAG	TCATTGTAGAGAATCAGGCGTGTTGAG
CDC25C	CCTCGAATGTGCCGTTCTCTGAG	AGCCTGGTGGTCCTGGTGAAG
Cdkn1a	TCCTGGTGATGTCCGACCTGTTC	CGGCGCAACTGCTCACTGTC
CCNA2	CACTGACACCTCTTGACTATCCAATGG	GTCTGGCTGCCTCTTCATGTAACC
CCNE1	CGTCCTGGATGTTGGCTGCTTAG	CTATGTCGCACCACTGATAACCTGAG
CCNB1	GCCTGAGCCTGAACCTGAACTTG	CATCATCTGCGTCTACGTCACTCAC
Cdkn1b	CGGATGGACGCCAGACAAGC	CTCTCCACCTCCTGCCACTCG
ECT2	CGACAGCGTGTTACCATCTCCTTC	ATCCTGGACCATTATCACTCTTGCTTC
ACTB1	ATCACTATTGGCAACGAGCGGTTC	CAGCACTGTGTTGGCATAGAGGTC

Supplementary Table III

mice	FORWARD	REVERSE
motif 1	ACAACTCCATTACTTTGTAAGCTAAGC	CAAGCTTGCTGTGAGCCGTC
motif 2	ATCTTGTTAGTTGATCAAGGTGTTTCA	TCTTGGTTTAGTTATACTGTACCATACC
rat		
motif 1	TCCTGTTTCAGCCAGTACTTCCA	AGAATTCAGGGAAGTGCTTCTAAAGA
motif 2	CCACAAGATGGCAGCAAACATT	TGATGTCACCAAATCTCTCAATGCA

Supplementary Table IV

	Normoxia	Hypoxia	P value
N	10	10	
CO (µl/min)	3367.55±114.33	1547.33±105.11	<0.0001
SV (µl)	16.28±0.44	7.95±0.54	<0.0001
Ves (µl)	3.89±0.92	6±0.14	<0.0001
Ved (µl)	19.43±0.48	12.34±0.47	<0.0001
HR (bpm)	206.85±4.08	194.76±1.3	<0.0001
EF (%)	84.76±2.91	70.85±6.52	<0.0001
Ea (mmHg/µl)	4.66±0.17	9.06±0.68	<0.0001

Supplementary Table V

	H3w+D1w	H3w+I1w	H3w+C1w	P value (H3w+C1w vs. H3w+D1w)	P value (H3w+C1w vs. H3w+I1w)
N	10	10	10		
CO (µl/min)	1262.33±76.86	678.89±71.92	1242.24±100.89	>0.05	<0.0001
SV (µl)	6.02±0.35	4.52±0.33	7.02±0.5	<0.0001	<0.0001
Ves (µl)	7.14±0.17	6.46±0.29	6.84±0.36	0.002	0.003
Ved (µl)	11.27±0.47	9.73±0.34	12.33±0.46	<0.0001	<0.0001
HR (bpm)	209.65±3.4	150.33±11.69	177.03±9.94	<0.0001	<0.0001
EF (%)	50.53±3.48	45.04±3.07	59.39±3.76	<0.0001	<0.0001
Ea (mmHg/µl)	11.74±0.69	13.22±1.03	8.31±0.49	<0.0001	<0.0001

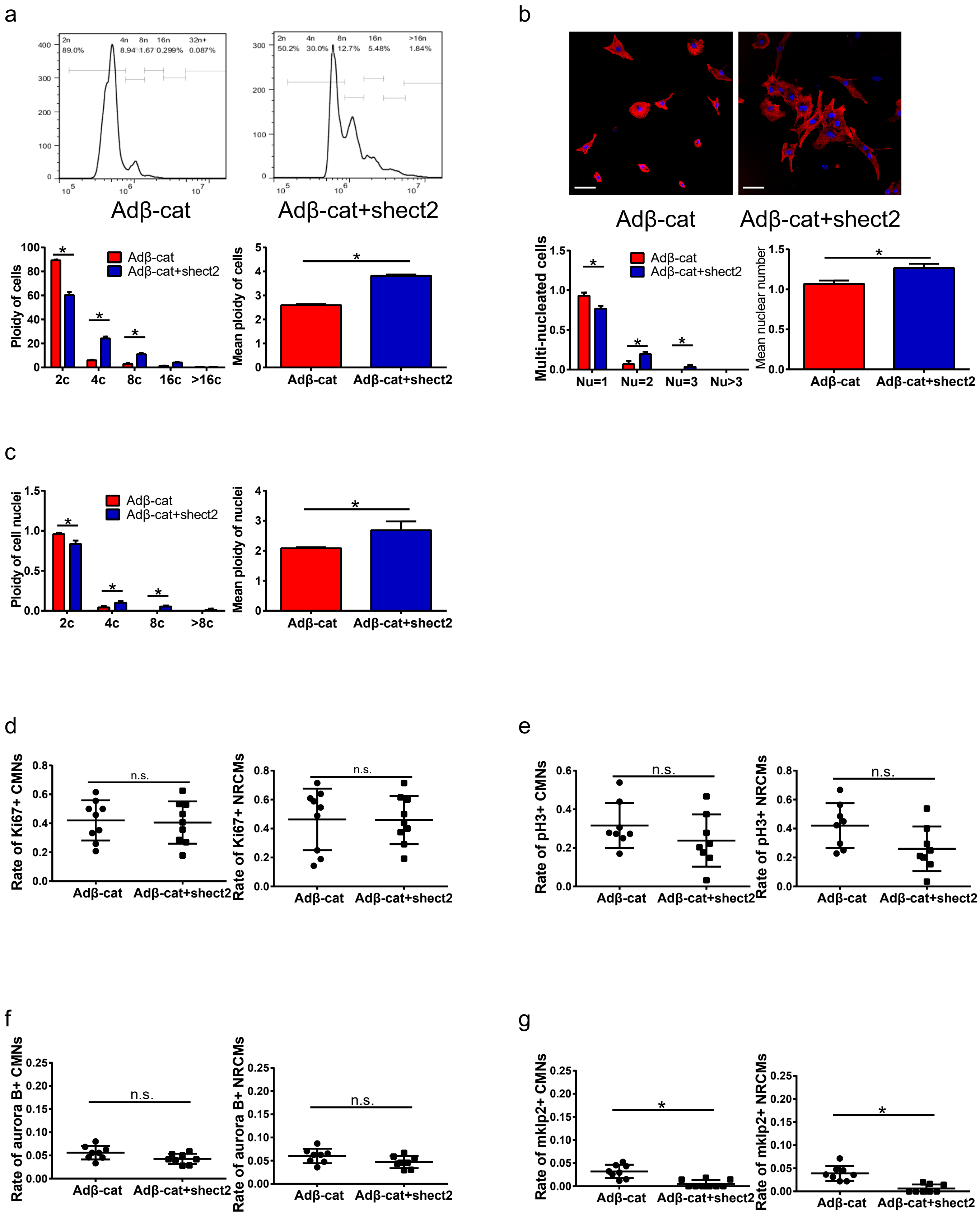

a
b
c
d
e
f
g
2n
89.0%
4n
8.94
8n
1.67
16n
0.299%
32n+
0.087%
50.2%
30.0%
12.7%
16n
5.48%
>16n
1.84%
Adβ-cat
Adβ-cat+shect2
Ploidy of cells
Mean ploidy of cells
2c
4c
8c
16c
>16c
Multi-nucleated cells
Mean nuclear number
Nu=1
Nu=2
Nu=3
Nu>3
Ploidy of cell nuclei
Mean ploidy of nuclei
>8c
Rate of Ki67+ CMNs
Rate of Ki67+ NRCMs
Rate of pH3+ CMNs
Rate of pH3+ NRCMs
Rate of aurora B+ CMNs
Rate of aurora B+ NRCMs
Rate of mklp2+ CMNs
Rate of mklp2+ NRCMs
n.s.
*
